# Limited Brain Metabolism Changes Differentiate between the Progression and Clearance of Rabies Virus

**DOI:** 10.1371/journal.pone.0087180

**Published:** 2014-04-24

**Authors:** Keith Schutsky, Carla Portocarrero, D. Craig Hooper, Bernhard Dietzschold, Milosz Faber

**Affiliations:** 1 Department of Microbiology and Immunology, Thomas Jefferson University, Philadelphia, Pennsylvania, United States of America; 2 Department of Cancer Biology, Thomas Jefferson University, Philadelphia, Pennsylvania, United States of America; University of Cincinnati School of Medicine, United States of America

## Abstract

Central nervous system (CNS) metabolic profiles were examined from rabies virus (RABV)-infected mice that were either mock-treated or received post-exposure treatment (PET) with a single dose of the live recombinant RABV vaccine TriGAS. CNS tissue harvested from mock-treated mice at middle and late stage infection revealed numerous changes in energy metabolites, neurotransmitters and stress hormones that correlated with replication levels of viral RNA. Although the large majority of these metabolic changes were completely absent in the brains of TriGAS-treated mice most likely due to the strong reduction in virus spread, TriGAS treatment resulted in the up-regulation of the expression of carnitine and several acylcarnitines, suggesting that these compounds are neuroprotective. The most striking change seen in mock-treated RABV-infected mice was a dramatic increase in brain and serum corticosterone levels, with the later becoming elevated before clinical signs or loss of body weight occurred. We speculate that the rise in corticosterone is part of a strategy of RABV to block the induction of immune responses that would otherwise interfere with its spread. In support of this concept, we show that pharmacological intervention to inhibit corticosterone biosynthesis, in the absence of vaccine treatment, significantly reduces the pathogenicity of RABV. Our results suggest that widespread metabolic changes, including hypothalamic-pituitary-adrenal axis activation, contribute to the pathogenesis of RABV and that preventing these alterations early in infection with PET or pharmacological blockade helps protect brain homeostasis, thereby reducing disease mortality.

## Introduction

Rabies is a central nervous system (CNS) disease caused by infection with rabies virus (RABV), a negative-stranded RNA virus of the *Rhabdoviridae* family. Despite the fact that rabies is one of the oldest known infectious diseases, it continues to present a veterinary and public health threat worldwide. Each year, more than 70,000 humans die from rabies around the globe with millions more undergoing post-exposure prophylaxis [1]. Rabies is a zoonotic disease and carnivores, especially dogs, are the main carriers of the virus [2]–[4]. RABV is usually transmitted via the bite of an infected animal. It is commonly understood that the virus travels from the site of exposure (e.g. open wound) through retrograde axonal transport into the CNS [5]–[7]. Once humans have developed clinical rabies there is currently no approved treatment owing to features of the infection that allow wild-type RABV to evade immune clearance from the CNS [7], [8]. Recently, live, highly attenuated rabies virus vaccines have been developed and shown to be efficacious in rabies post-exposure treatment of (PET) animal models [9]. The recombinant RABV TriGAS is one such promising vaccine candidate, with an excellent pre- and post-exposure therapy profile. It is highly immunogenic and nonpathogenic for mice that are either developmentally immunocompromised or have inherited deficits in immune function as well as normal adult animals even when administered intracranially [9]–[11]. TriGAS is capable of inducing both the immune mechanisms and effectors that promote their infiltration into CNS tissues resulting in containment and clearance of a pathogenic RABV CNS infection [10]. However, the mechanisms underlying rabies pathogenesis as well as host responses that are able to restrict and eventually clear the infection are likely complex and not fully understood. Notably, infection of the CNS with wild-type (WT) RABV does not result in neuronal loss or overt neuronal damage [12]–[14]. Nonetheless, a significant factor responsible for the lethal outcome of an RABV infection appears to be neuronal dysfunction due to drastically inhibited synthesis of proteins required in maintaining basic cellular and metabolic functions [15]. Another characteristic of rabies is the limited immune cell infiltration into the CNS tissues seen in human patients and rodents infected with pathogenic RABV, which led to the concept that RABV has the ability to evade immune clearance [16], [17].

To obtain further insight into mechanisms involved in RABV pathogenesis and the host responses that can prevent the lethal outcome of the infection, we recently compared the transcriptomes of uninfected mouse brain with those of WT RABV-infected mice that had received either mock treatment or TriGAS PET. This analysis showed that genes that play key roles in adaptive immunity (e.g. Ccl3, IL12B, GzmA) are activated earlier and to a greater extent in RABV-infected, TriGAS-treated mouse brains versus RABV-infected, mock treated brains suggesting that timely activation of these genes during the infection is likely an important part of the mechanism by which TriGAS mediates its protective activity [10]. However, the conclusions that can be drawn from transcriptome analyses are limited since mRNA expression data does not reveal the numerous biochemical processes in the brain that are likely altered during the course of infection. There is now growing evidence that metabolic profiling of living systems can provide meaningful information about perturbations of metabolism caused by genetic deficits, aging or exogenous factors thought to occur in different disease processes [18], [19]. Hence, we utilized metabolomics to characterize brain metabolism associated with rabies infection and post-infection viral clearance facilitated by TriGAS vaccine treatment. This analysis revealed significant changes in biochemicals associated with energy metabolism in infected, mock treated mice that were largely prevented with vaccine treatment. Mitigation of changes in neurotransmitters and stress hormones due to vaccine treatment may be predictive of successful post-exposure therapy and, additionally, suggest new possibilities for therapeutic intervention. In support of this concept, we show that pharmacological intervention to inhibit corticosterone biosynthesis, in the absence of vaccine treatment, significantly reduces the pathogenicity of wild-type RABV strain DOG4.

## Results

### PET with TriGAS reduces virus load and inhibits viral spread in the CNS of mice infected with DOG4 RABV

**Figure 1:**
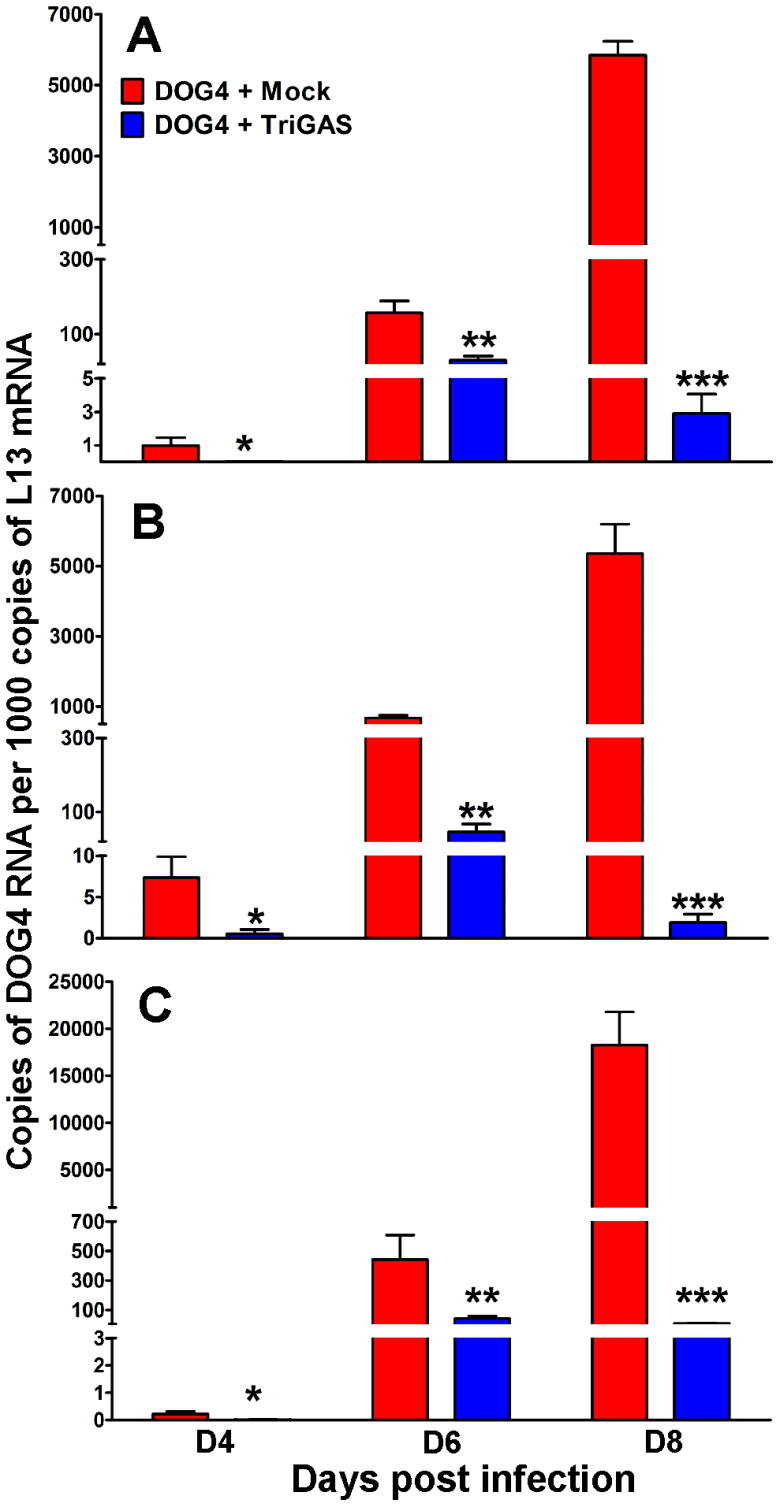
Post-exposure treatment of DOG4 RABV-infected mice with TriGAS dramatically reduces the accumulation of RABV N protein mRNA in the brain. Mice were infected i.n. with 10^5^ FFU of DOG4 RV and 4 hr later either mock treated with PBS or treated with 10^7^ FFU of TriGAS. Ten to 15 mice per group were euthanized for each tissue at each indicated time point, and the number of DOG4 RABV N mRNA copies in A) whole brain, B) olfactory bulb and C) hypothalamus was quantified by qRT-PCR as described in Materials and Methods. The results are presented as the mean RABV N mRNA copy numbers (+SE) per 1,000 copies of L13 mRNA. The bars indicate significant differences in copy numbers between mock-treated and PET-treated animals. *p<.05, **p<.01 and ***p<.001, respectively.

To correlate changes in brain metabolites with RABV infection of the brain, we first utilized RT-qPCR analysis to determine the transcription levels of DOG4 RNA in the CNS of mock- and TriGAS-treated DOG4-infected mice at 4, 6, and 8 days post inoculation (p.i.). As shown in Figure 1A–C, DOG4 RABV-specific RNA is detected in whole brain, olfactory bulb and hypothalamus of mock and TriGAS-treated mice at 4 days after infection, although normalized DOG4 RNA copies were significantly lower in the brain regions of vaccine-treated mice (0.98, 7.34, and 0.22 copies in mock-treated group vs. 0.03, 0.56, 0.01 copies in vaccine-treated group; p<.05 each region, respectively). By 6 days p.i, DOG4 RNA levels increased in the respective brain tissues of both groups of mice, although levels in whole brain, olfactory bulb, and hypothalamus were significantly lower in vaccine versus mock-treated animals (30, 46, 43 copies vaccine vs. 157, 669, 442 copies mock; p<.01 each region). Importantly, while DOG4 RNA levels in all brain regions of mock-treated animals dramatically increased at day 8 p.i, they declined significantly at this time in TriGAS-treated mice (583, 5358, 18252 copies mock vs. 2.9, 1.9, 8.5 copies vaccinated; p<.001 each region).

### Changes in levels of brain metabolites associated with progression or inhibition of RABV infection

After cardiac perfusion and placement of each brain in methanol, a total of 259 named biochemicals were analyzed in CNS samples of 1) uninfected, untreated control, 2) infected, mock-treated, and 3) infected, vaccine-treated animals taken at 0, 4, 6, and 8 days post-infection (p.i.) using liquid chromatography/mass spectrometry (Table 1). Following log transformation and imputation with minimum observed values for each compound, Welch's two-sample *t*-test was used to identify biochemicals that differed significantly between experimental groups. A summary of the number of changes in the levels of biochemicals that achieved statistical significance (*p*≤0.05), as well as those approaching significance (0.05<*p*<0.1) is shown in Table 1.

**Table 1:** Significantly altered biochemicals during the course of WT RABV infection in the presence or absence of PET with TriGAS.

Significantly Altered Biochemicals (Welch's Two Sample t Test)	Mock Treatment vs. Uninfected Control.			Vaccine Treatment vs. Uninfected Control			Vaccine Treatment vs. Mock Treatment		
Days post infection	Day 4	Day 6	Day 8	Day 4	Day 6	Day 8	Day 4	Day 6	Day 8
Total biochemicals p≤0.05	30	41	54	21	26	7	8	8	69
Biochemicals (↑↓)	20|10	16|25	27|27	13|8	5|21	48|22	3|5	2|6	52|17
Total biochemicals 0.05<p<0.10	20	23	16	25	27	26	9	15	25
Biochemicals (↑↓)	13|7	12|11	9|7	22|3	12|15	15|11	4|5	6|9	15|10

Summarized is the number of metabolites significantly altered during early (D4), middle (D6) and late-stage WT RABV infection (D8) with or without vaccine as well as the amount of those that approach significance.

### The majority of alterations in brain metabolism occur late in infection

While the results of the metabolome analysis demonstrated a limited number of differences between vaccine and mock-treated mice infected with RABV at days 4 and 6 post infection, there were a much larger number of statistically significant changes at day 8. Principal Component Analysis (PCA) was used to investigate associations between metabolite levels and infection/treatment. This approach transforms a large number of metabolic variables into a smaller number of orthogonal variables (PC1, PC2, PC3, etc.), represented as symbols on a plot, that allow differences between separate conditions to be assessed based on clustering. Using such an approach, we found that biochemicals tended to cluster together according to time and treatment with the exception of the day 8 samples (Figure S1), which differed dramatically between the three groups. While the number of statistically significant changes was low prior to the day 6 time point (Table 1), at day 8 p.i. vaccine and mock treatment resulted in a multitude of changes as shown in the heat map (Table S1) and the PCA plot (Figure S1), which distinguishes the results at this time point from those of the earlier time points. The largest differences between vaccine and mock treatment during the course of the study were seen in the levels of acetylcholine (Ach), energy metabolites, carnitines and carnitine-related metabolites, and corticosterone.

### PET with TriGAS prevents the infection-induced reduction in Ach levels

**Figure 2:**
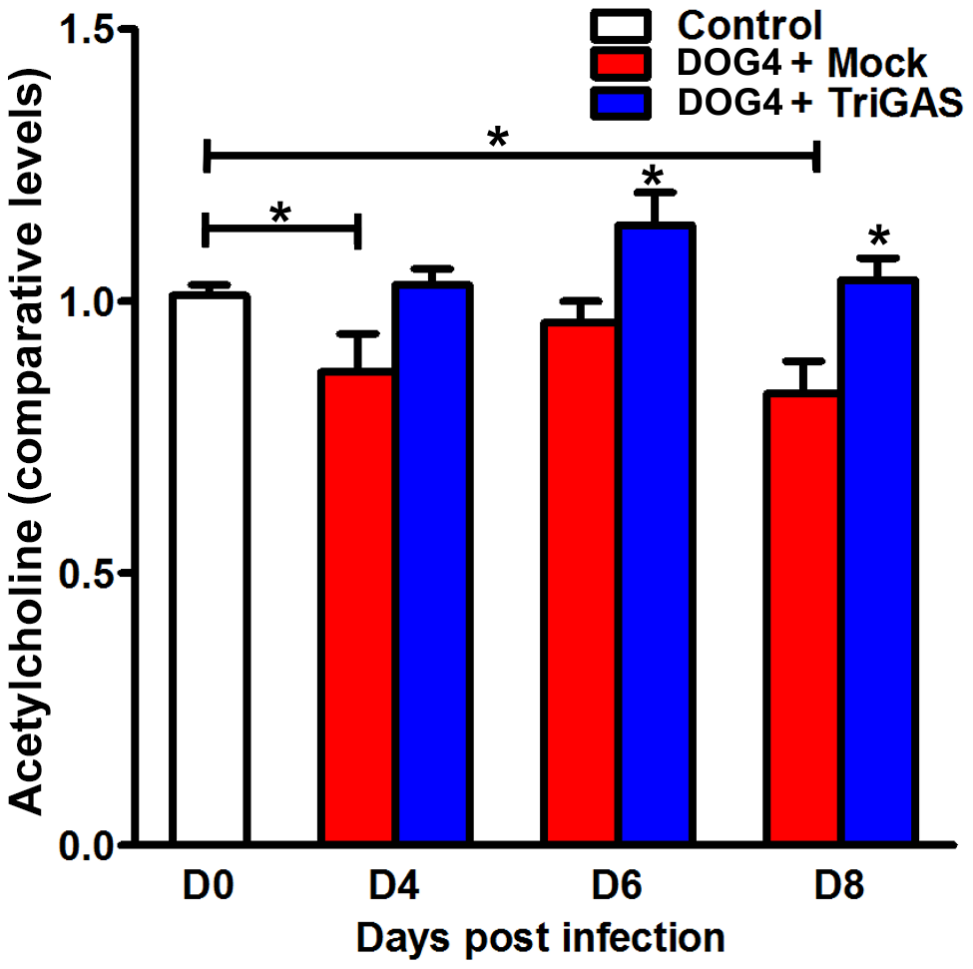
PET with TriGAS prevents the RABV infection-induced decline in acetylcholine. Mice were infected i.n. with 10^5^ FFU of DOG4 and 4 hr later either mock treated i.m. with PBS or treated with 10^7^ FFU of TriGAS. An additional group of uninfected, untreated mice served as a reference. Six mice per group were euthanized for each time point, and the levels of acetylcholine were determined via LC-GC/MS-MS2 as described in Materials and Methods. The results are presented as comparative levels (+/− SE). Statistical differences between groups were analyzed using Welch's two sample t test and are identified by *p<0.05. The horizontal lines represent significant differences between control and DOG4-infected/mock-treated mice while the asterisks over bars represent differences between mock- and TriGAS –treated, infected mice.

We found Ach to be significantly lower at days 6 and 8 post-infection in the CNS tissues of mice that received mock treatment by comparison with similarly infected mice that received vaccine (Figure 2). There was an increase in the disparity between the two groups over time (15.1+/−7.1% D4, 15.8+/−3.7% D6, & 19.5%+/−5.5%). Furthermore, there were also significant differences in Ach levels between the mock-treated and uninfected, untreated animals at D4 and D8 p.i, p<.05. However, no significant differences in acetylcholine levels were detected between the CNS tissues of normal versus DOG4/TriGAS-treated animals. The declining Ach levels during the course of RABV infection likely result in progressively impaired cholinergic neurotransmission [20], [21].

### RABV infection elevates brain energy metabolites

**Figure 3:**
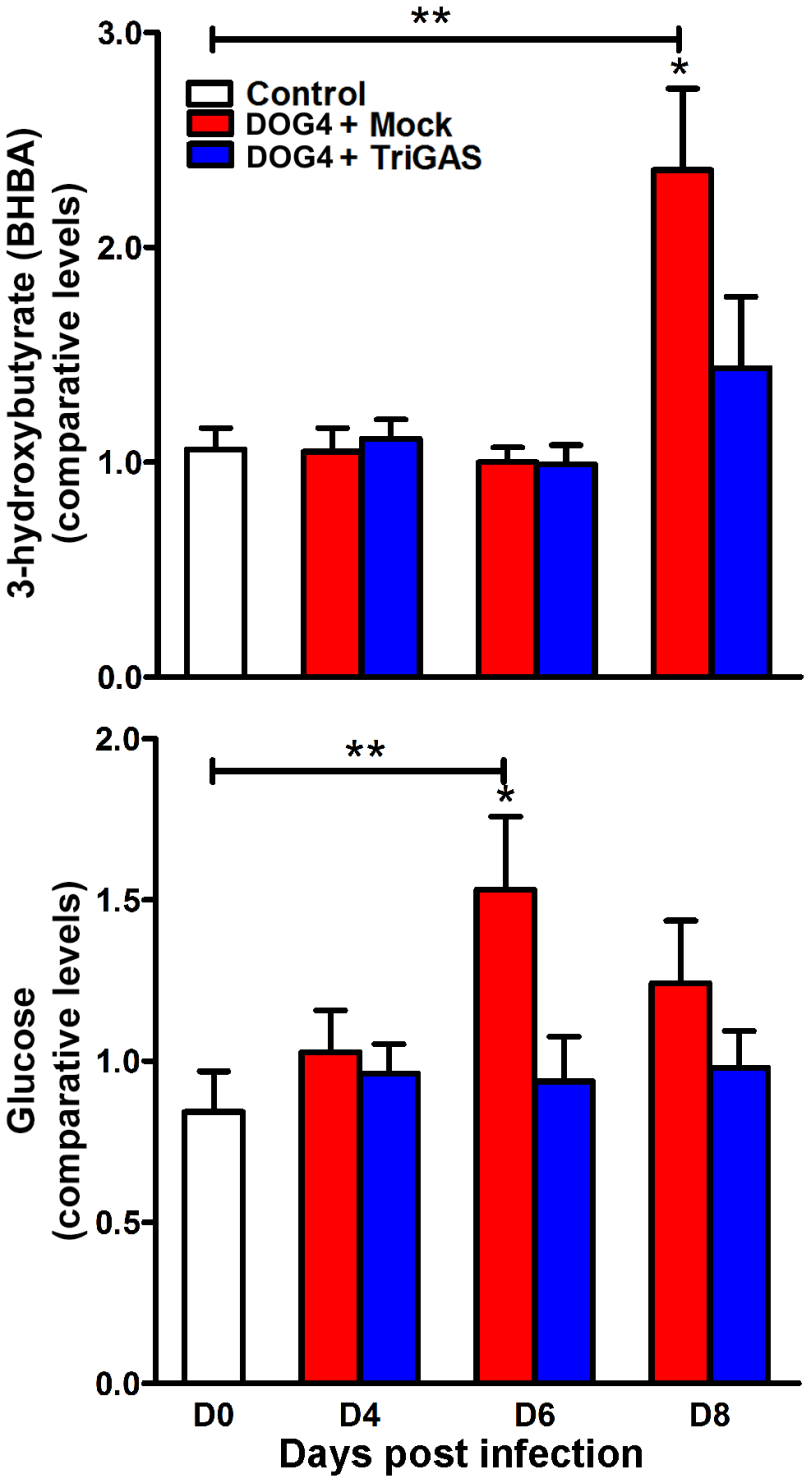
Energy metabolites 3-hydroxybutyrate (BHBA) and glucose increase in the CNS during the course of WT RABV infection. Mice were infected i.n. with 10^5^ FFU of DOG4 and 4 hr later either mock treated i.m. with PBS or treated with 10^7^ FFU of TriGAS. An additional group of uninfected, untreated mice served as a reference. Six mice per group were euthanized for each time point, and the levels of BHBA and glucose were determined via LC-GC/MS-MS2 as described in Materials and Methods. The results are presented as comparative levels (+/− SE). *p<.05, **p<.01.

Pathogenic RABV-infected, mock-treated animals displayed significantly higher levels of the ketone body 3-hydroxybutyrate (BHBA) at day 8 in relation to the vaccine-treated samples in this study (Figure 3A), p<.05. Notably, mock-treated animals also exhibited a 123+/−36% increase in BHBA in relation to uninfected controls at this time (p<.01). Furthermore, there was a significant increase in glucose levels in mock-treated mice in relation to vaccine treated RABV-infected mice at day 6 (Figure 3B), p<.05, with mock-treated animals also displaying a significant increase over uninfected, untreated controls at this time (p<.01). The increase in both of these energy-associated metabolites in the brain tissue of mock-treated mice may be an indication of decreased glucose utilization or increased glucose uptake by the brain.

### Carnitine and carnitine-related metabolites are increased by vaccine treatment

**Figure 4:**
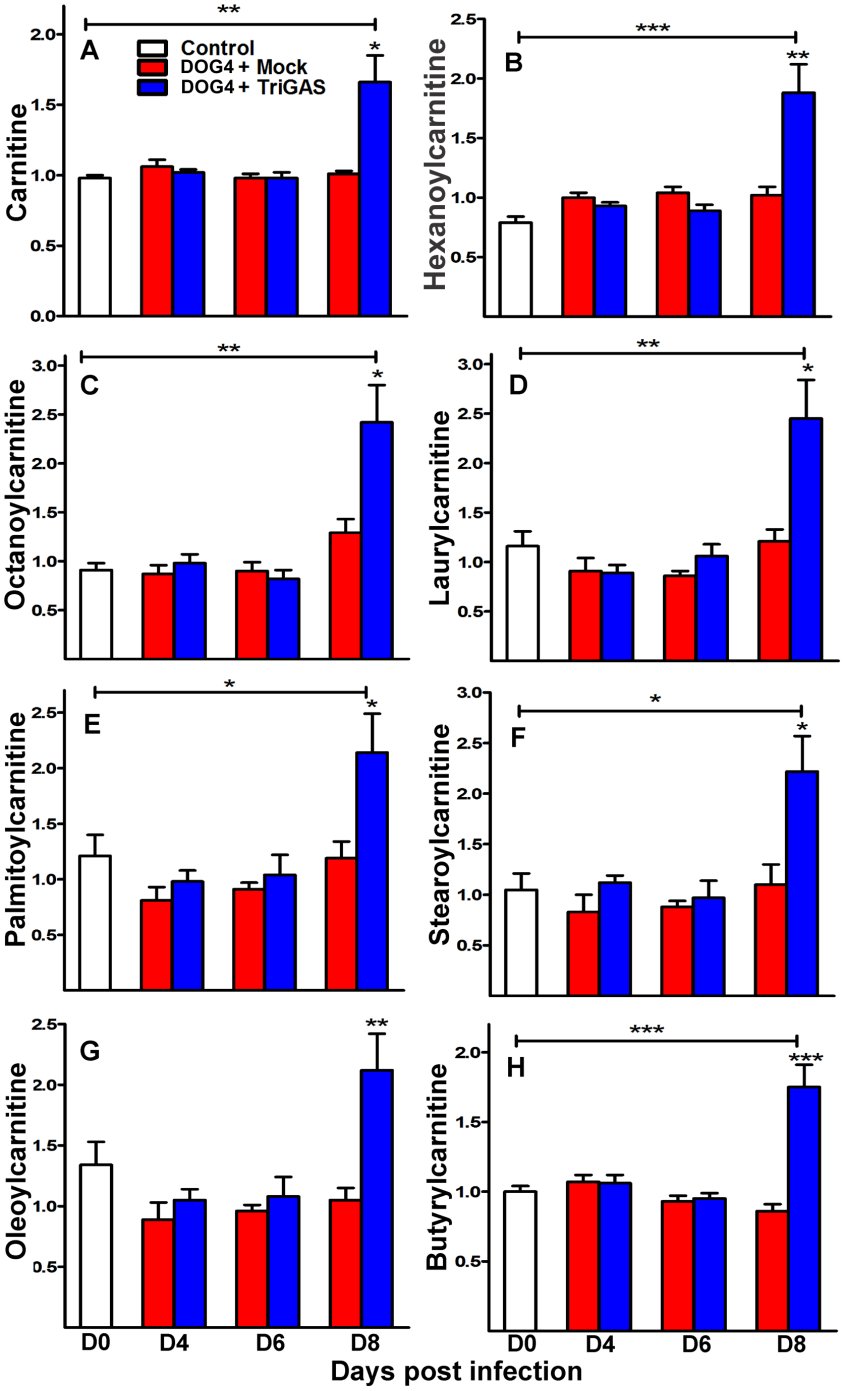
Levels of carnitine and several acylcarnitines increase in the brains of TriGAS treated mice late in RABV infection. Mice were infected i.n. with 10^5^ FFU of DOG4 and 4 hr later either mock treated i.m. with PBS or treated with 10^7^ FFU of TriGAS. An additional group of uninfected, untreated mice served as a reference. Six mice per group were euthanized for each time point, and the levels of carnitine (A) and acylcarnitines: hexanoylcarnitine (B), octanoylcarnitine (C), laurylcarnitine (D), palmitoylcarnitine (E), stearoylcarnitine (F), oleoylcarnitine (G), and butyrylcarnitine (H) were determined via LC-GC/MS-MS2 as described in Materials and Methods. The results are presented as comparative levels (+/− SE). *p<.05, **p<.01, ***p<.001.

One interesting finding in this study was that carnitine and a number of acylcarnitines (hexanoylcarnitine, octanoylcarnitine, laurylcarnitine, palmitoylcarnitine, stearoylcarnitine, oleoylcarnitine and butyrylcarnitine), were significantly higher at day 8 p.i. in the brains of vaccine-treated mice in relation to either the uninfected controls or mock-treated mice (Figure 4A–H), (*p<.05 - *** p<.001). The increase in carnitine levels could be one of the mechanisms by which TriGAS exerts its protective activity against a lethal RABV infection. These biochemicals have been shown to be protective in animal models of ischemia, neurodegeneration and viral-induced encephalitis [22], [23].

### Levels of corticosterone are higher in mock-treated mice than in vaccine-treated mice

**Figure 5:**
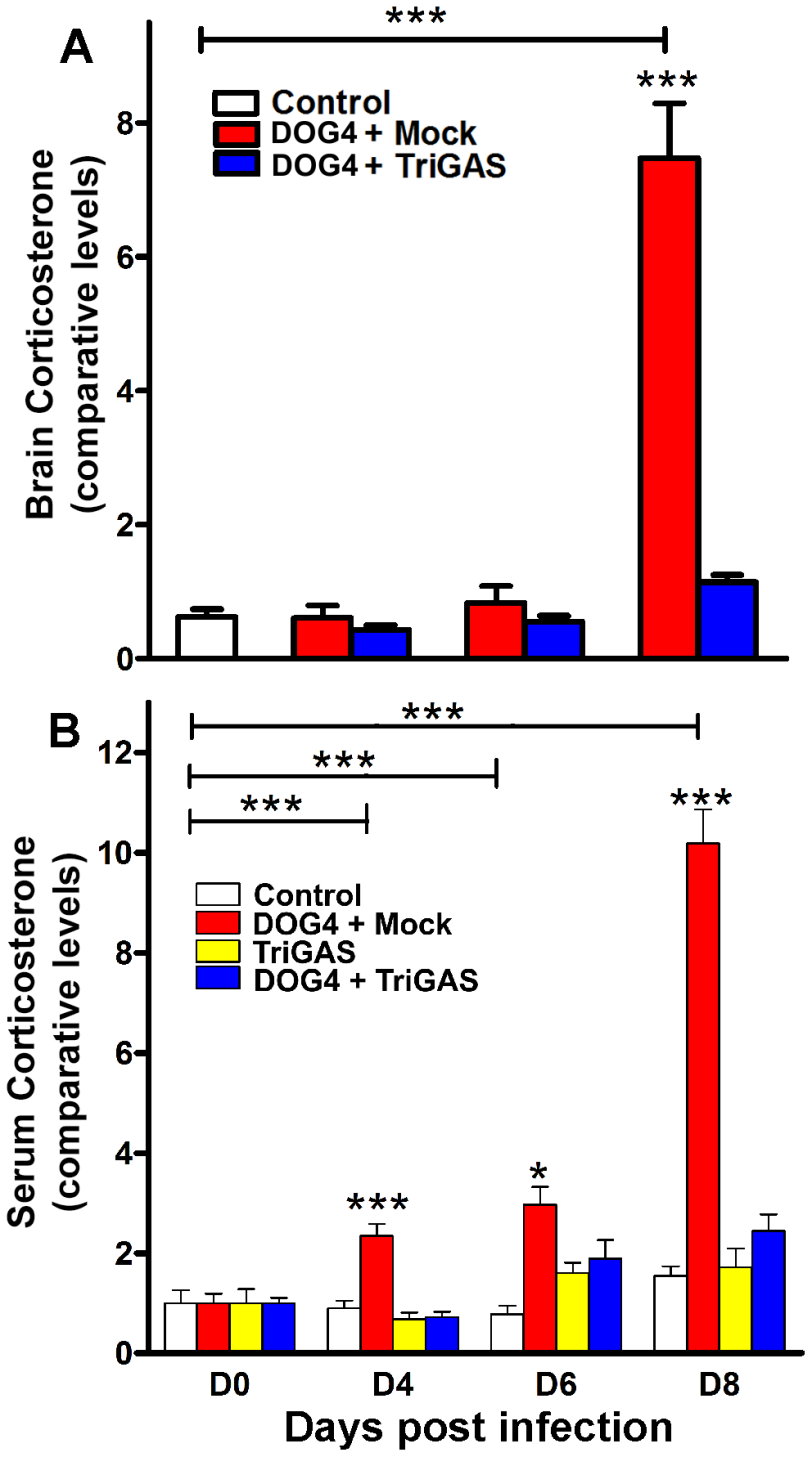
Postexposure treatment of DOG4 RV-infected mice with TriGAS prevents the elevation of corticosterone associated with pathogenic RABV in brain and serum. A) Mice were infected i.n. with 10^5^ FFU of DOG4 and 4 hr later either mock treated with PBS or treated with 10^7^ FFU of TriGAS. An additional group of uninfected, untreated mice served as a reference. Six mice per group were euthanized for each time point, and the levels of brain corticosterone were determined via LC-GC/MS-MS2 as described in Materials and Methods. B) Groups were identical to those in A), with one additional group of animals receiving only TriGAS. Ten mice per group were bled at each time point and serum corticosterone was measured by EIA as described in Materials and Methods. The results represent comparative levels of corticosterone (+/− SE) *p<.05, **p<.01, and ***p<.001, respectively.

The most striking change in this study was a 12-fold higher level of corticosterone at day 8 p.i. in mock-treated DOG4-infected mice versus uninfected controls, p<.001 (Figure 5A). Notably, vaccine treatment completely eliminated the rise in brain corticosterone associated with RABV infection. Corticosterone, a stress hormone produced by the adrenal cortex, has been reported to affect immune function and cellular metabolism and is regulated in large part, by the hypothalamic-pituitary axis (HPA) [24], which has been shown to be activated during RABV infection [25], [26]. Interestingly, at days 4 and 6 p.i, there were no significant differences in brain corticosterone levels between mock and vaccine treatment. It is possible that the surge in brain corticosterone at day 8 of infection is associated with the dramatic increase in virus replication in the hypothalamus and the adrenals of mock-treated mice seen at this time point (Figures 1, ). This conclusion is further supported by the finding that the significantly lower corticosterone levels in the TriGAS-treated mice correlate with limited CNS infection at day 8 p.i. (Figure 1A–C).

### Serum corticosterone levels are elevated in mock-treated mice early after RABV infection and continue to escalate as the infection proceeds

Since corticosterone is produced in the periphery, we investigated whether serum levels of corticosterone were higher in infected, mock-treated animals at earlier times than in brain tissue. Indeed, we found serum corticosterone levels were significantly elevated in mock-treated animals as early as 4 days p.i., and continued to climb during the course of infection, reaching a pinnacle by day 8 (Figure 5B). Importantly, vaccine treatment, either by itself or with infection, caused no significant elevation in serum corticosterone at any time during the study. To control for stress effects induced from repeatedly bleeding each group of animals, corticosterone was measured in uninfected, mock-treated mice prior to treatment and at days 4, 6, and 8 afterwards. Notably, corticosterone levels did not significantly differ at any any time point in these uninfected mock-treated animals. These results suggest that HPA activation occurs rapidly after RABV infection, where corticosterone accumulates in the periphery and then enters the CNS.

### Post-exposure therapy with metyrapone, a corticosterone synthesis inhibitor, reduces serum levels of corticosterone in infected mice and attenuates the pathogenicity of RABV

**Figure 6:**
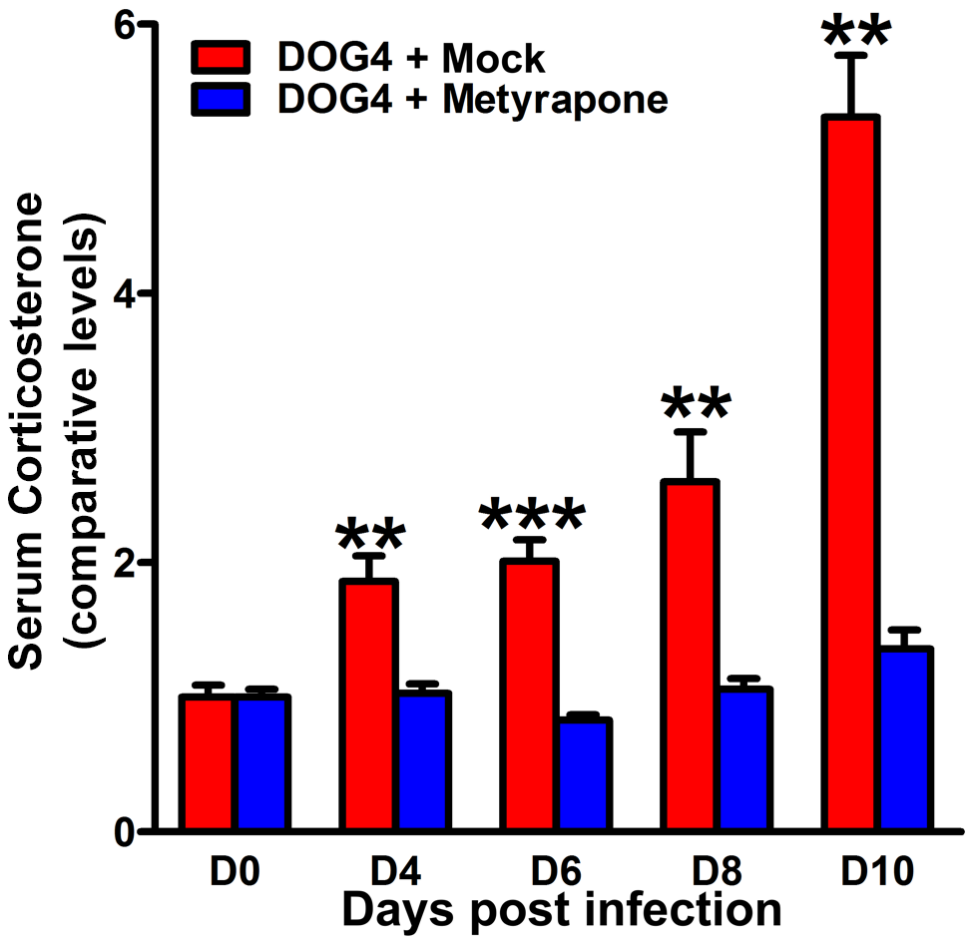
Post-exposure treatment of DOG4 RV-infected mice with metyrapone blocks the rise in corticosterone associated with pathogenic RABV. Mice were infected with 10^4^ FFU of DOG4 RABV i.n. and either mock treated with PBS or treated with metyrapone (100 mg/kg) i.p. each day for 21 days. Ten mice per group were bled 1 hr after injection for each time point. The results represent comparative levels of corticosterone (+/− SE). *p<.05, **p<.01, and ***p<.001, respectively.

**Figure 7:**
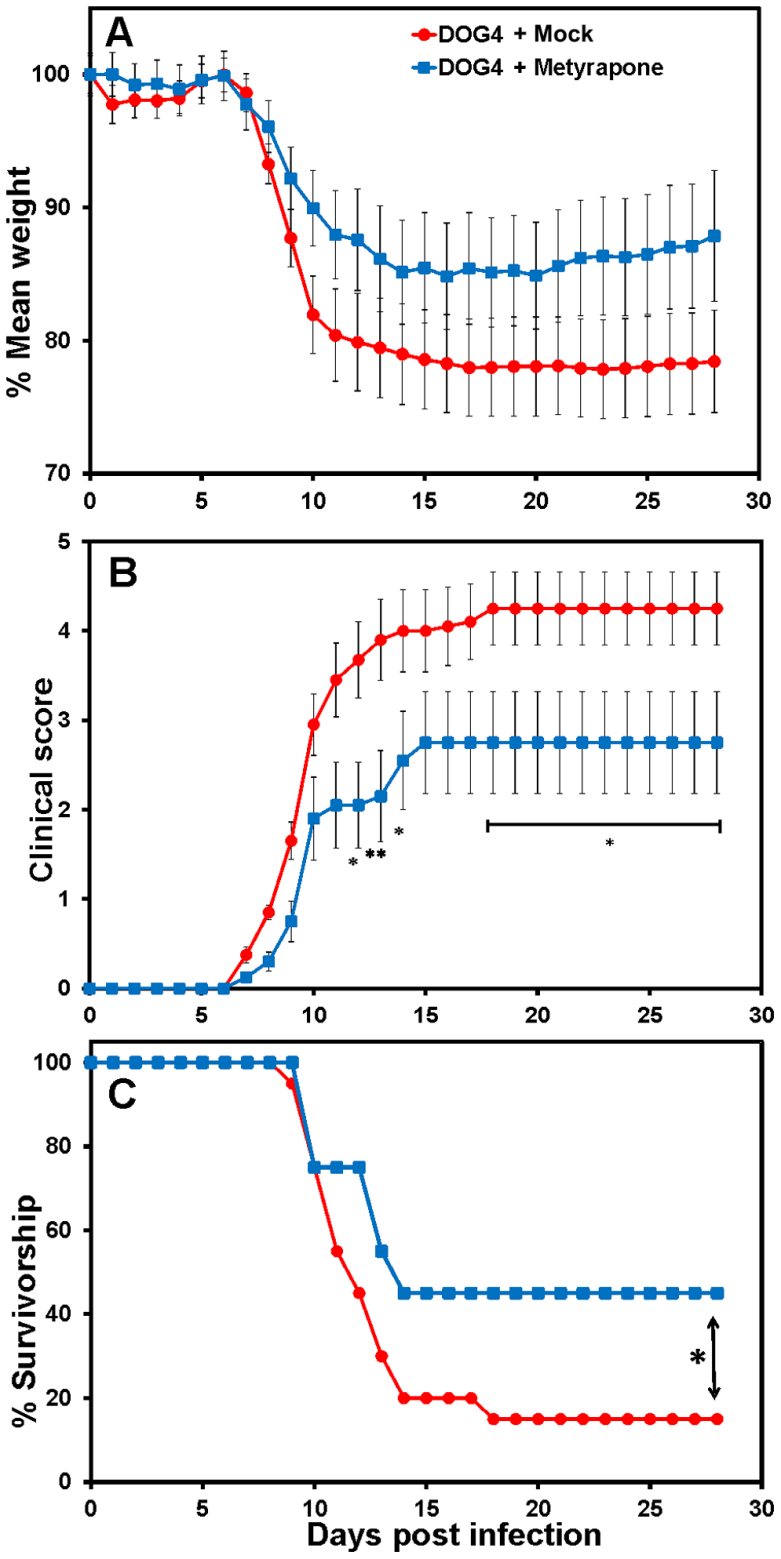
PET with metyrapone, a corticosterone synthesis inhibitor, reduces the pathogenicity of WT RABV. Groups of 20 mice were infected with 10^4^ FFU of DOG4 RABV i.n. and either mock-treated with PBS or treated with metyrapone (100 mg/kg) i.p. each day for 21 days. The mice were observed for 4 weeks, and body weight (A), clinical signs (B) and survival (C) are presented (+/− SE). For (A), two-way anova was used to determine significance effects in treatment per day. (B) Two-way anova and Bonferroni post hoc tests were used to indicate daily differences in clinical severity (*p<.05, **p<.01). (C) The Mantel-Cox log-rank test was used to determine differences in survivorship (*p<.05).

Dramatically elevated levels of brain and serum corticosterone associated with RABV infection (Figure 5A–B) suggest that the production of corticosterone and its widespread effects on both immune function and cellular metabolism contribute to rabies pathogenesis. To test this hypothesis, we investigated whether treatment of RABV-infected mice i.p. with metyrapone, which inhibits the conversion of 11-deoxycortisol to corticosterone in the terminal reaction of the glucocorticoid biosynthetic pathway, affects the outcome of the infection. Metyrapone significantly attenuated the elevation in corticosterone associated with pathogenic RABV infection when administered each day for 21 days (beginning 4 hours post-infection) (Figure 6). The discrepancy between serum corticosterone levels of mock-treated DOG4-infected mice at day 8 p.i. shown in Figures 5B and 6 is likely due to the different challenge doses that were used in the two experiments. While mice in Fig. 5B were challenged with 10^5^ FFU of DOG4, mice in Figure 6 were challenged with only 10^4^ FFU of DOG4 which likely resulted in a delay of the disease process and thus a shift in the corticosterone production. Strikingly, metyrapone treatment ameliorated body weight loss and clinical signs associated with infection, leading to significantly reduced mortality (Figure 7A–C). Metyrapone treatment had no effect on virus replication in the CNS over the first critical 8 days of infection (Table S2). Furthermore, comparison of the induction of RABV-specific antibody in metyrapone versus mock-treated infected animals over the first 8 days of infection (after which untreated infection is terminal) revealed no significant differences between the groups. However, the metyrapone-treated mice that survived DOG4 infection had developed significant serum VNA titers by day 12 p.i. (Table S3). The most likely explanation for the VNA titer increase in metyrapone treated mice after day 8 p.i. is that in the initial phase of the infection (up to day 8) the virus spread is largely confined to the brain thereby preventing the induction of antibody responses. During the later phase of the infection virus spreads into peripheral tissues where an immune response can be triggered.

## Discussion

Metabolome analysis of brain tissue from mock- and vaccine-treated RABV-infected mice was utilized to identify potentially important biochemicals associated with lethal infection or successful post-exposure prophylaxis with the live, highly attenuated RABV variant TriGAS. A similar approach has been used to elucidate biochemicals and pathways contributing influenza- and HIV-associated encephalopathies [27], [28]. In addition, metabolomics have been recently utilized to characterize alterations in the cerebral spinal fluid of human patients infected with rabies and in the postmortem CNS tissues of an individual who succumbed to the disease [29], [30]. The limitations of these studies are small sample sizes, the assessment of a restricted number of metabolites and the analysis being post mortem or limited to the late stages of the disease. Using an animal model we were able to examine changes in a large number of metabolites during the course of lethal rabies infection and contrast these with metabolic changes associated with a novel treatment modality.

Our study showed that levels of neurotransmitters, energy metabolites and stress hormones were altered during the course of WT RABV infection with the number of statistically significant changes accumulating as viral load in the CNS increased (Table 1, Figure 1A–C). This is consistent with reports demonstrating manifold functional and structural changes in neurons infected with rabies [31]–[33].

Notably, mock-treated animals displayed a marked reduction in Ach levels during the course of infection (Figure 2), which likely occurs via one or more mechanisms. The virus may be infecting brain regions involved in Ach production, including structures of the basal forebrain, or it may reduce the activity of choline acetyltransferase, the enzyme involved in Ach synthesis. Alternatively, RABV may be changing the activity of the vesicular Ach transporter affecting how the neurotransmitter is stored and released or it may be increasing acetylcholinesterase activity, promoting the degradation of Ach. Nevertheless, there is evidence that the acetylcholine receptor interacts with RABV, facilitating rabies virus entry into specific neurons [20], [21]. Notably, Ach neurotransmission is impaired in other encephalopathies (e.g. Alzheimer's, Parkinson's) and acetylcholinesterase inhibitors which restore levels of Ach provide therapeutic benefit, at least initially [34]. If administered during rabies infection, such agents may prove efficacious by either improving cholinergic signaling or by promoting more effective competition between Ach and RABV at the receptor level, thereby reducing viral entry and spread. In support of this, it has been demonstrated that PET with mecamylamine, a non-competitive acetylcholine receptor antagonist, reduces mortality in C57BL6 mice infected with a moderately pathogenic strain of RABV [35].

Cellular metabolism of amino acids assists in the formation of neurotransmitters including Ach and our results suggest that rabies is, in part, a disease of altered CNS metabolism. Significantly elevated levels of glucose as well as the ketone body BHBA were associated with later stages of RABV infection (Figure 3). Increases in these important brain energy metabolites may signify decreased usage or increased uptake by the RABV-infected brain as it struggles to maintain cellular homeostasis in the face of a continuously increasing viral load. The lack of a significance difference in brain glucose levels between mock-treated, infected and vaccine-treated, infected animals at day 8 p.i. is not surprising as the mock-treated animals cease eating by this time and become hypoglycemic (Figure S3). Our results support accumulating evidence that rabies as well as many other CNS pathologies (e.g. Alzheimer's, Huntington's, HIV encephalitis) can be considered metabolic diseases as they result in progressive impairment of the brain's capacity to utilize glucose and/or insulin [36]–[38]. Importantly, alterations in brain energy metabolism associated with RABV infection are fully ameliorated by vaccine treatment. Our results suggest that therapeutic interventions which help maintain metabolic function in the CNS (or conserve the rate of glucose utilization) could also be beneficial. Perhaps this is why induction of coma in humans infected with rabies decreases the rate of mortality [39].

The largest change found in this study was dramatically higher brain and serum corticosterone levels associated with infection in the absence of PET or pharmacological intervention, with serum levels increasing in infected animals before significant amounts of virus became detectable in the brain and clinical signs of the infection appeared (Figures 1, 5A–B, 7). The rise in corticosterone is unlikely to be exclusively the result of a stress response to RABV-mediated CNS damage because mock-treated mice exhibit no clinical signs or weight loss at 4 and 6 days p.i. when serum corticosterone levels are already elevated. It is more probable that corticosterone blocks the induction of inflammatory processes that promote immune cell activation, migration and infiltration into the CNS. Glucocorticoids, including corticosterone, have potent anti-inflammatory effects and reduce the expression of proinflammatory cytokines (TNF-alpha, IL-1), chemokines (CCL2, CXCL10), prostaglandins (COX-2) and inducible nitric oxide (iNOS) [40], [41]. Glucocorticoids also interfere with the ability of monocytes and dendritic cells to present antigen to T cells, reduce immune cell proliferation and promote apoptosis in leukocytes and T lymphocytes [42]. Moreover, it has been shown that glucocorticoid production after activation of the HPA axis can impair the innate immune response, promoting the development of certain infections [43]. In addition, several viruses including herpes viruses, cytomegalovirus and New Castle disease virus have been shown to activate the HPA axis [44]. Steroid hormones have been demonstrated to reduce blood-brain barrier permeability and immune effector infiltration into CNS tissues in mice infected with attenuated RABV leading to increased mortality [17]. Similarly, administration of corticosteroids has been shown to hasten the rate of RABV mortality and morbidity in multiple animal species [25]. Humans infected with rabies display high levels of cortisol [45], and elevated levels of this hormone, because of its early induction, may serve as a biomarker for disease progression. Interestingly, while treatment with metyrapone, an inhibitor of corticosterone biosynthesis, prevented the increase in serum corticosterone levels and reduced the pathogenicity of DOG4 infection (Figure 7A–C), it did not reduce virus loads in the brain over the critical first 8 days of infection. Since the predominant effect of corticosteroids is anti-inflammatory, we speculate that metyrapone promotes pro-inflammatory mechanisms, which have been shown to be necessary for the antibody-mediated protection against a rabies virus infection [46].

PET with TriGAS eliminated the large majority of neurological abnormalities associated with WT RABV infection, including alterations in levels of Ach, energy metabolites and corticosterone (Figures 2, 3, 5A–B). TriGAS likely mediates these effects by reducing viral replication levels in the CNS (Figure 1A–C) and by promoting the rapid development of innate and adaptive immunity [11], [47]. Interestingly, carnitine and acylcarnitine derivatives were significantly upregulated in treated compared to mock-treated RABV-infected animals. Carnitines have been shown to support immune function, supporting the production of CD4+ and CD8+ T cells during infection [48], [49]. Interestingly, the time-dependent rise in carnitine levels shown in infected mice that were administered TriGAS correlates with the development of adaptive immunity, including extensive B and T cell infiltration into the CNS at 8 days p.i. [47]. In addition to their immunomodulatory effects, carnitines have been shown to enhance Ach neurotransmission [50], preserve mitochondria function [51] and promote lipid synthesis, all of which may be mechanisms of neuroprotection [52].

In total, our results suggest that widespread metabolic changes, including hypothalamic-pituitary-adrenal axis activation, are key contributors to the pathogenicity of WT RABV and that preventing these alterations early in infection with PET or pharmacological blockade may help protect brain homeostasis and allow for the development of the adaptive immune mechanisms necessary to clear an otherwise lethal infection.

## Materials and Methods

### Viruses

The recombinant RABV SPBAANGAS-GAS-GAS (TriGAS) was generated as described elsewhere [9] and propagated in BSR cells, a derivative of BHK-21 cells [53]. The pathogenic WT RABV strain DOG4 was propagated in NA cells (C1300 mouse neuroblastoma, clone NA) as described previously [9]. To determine virus titers, NA cells were grown for 2 days, and the monolayers were infected with virus in 10-fold serial dilutions. Forty-eight hours postinfection (p.i.), the cells were fixed with 80% acetone and stained with fluorescein isothiocyanate (FITC)-labeled RABV nucleoprotein (N)-specific antibody (Fujirebio Diagnostics, Malvern, PA). Virus titers in triplicate samples were determined by counting foci of infected cells using a fluorescence microscope and are expressed as focus forming units (FFU) [54].

### Real-time qPCR analysis to determine viral replication *in vivo*

Eight- to 10-week-old female Swiss Webster mice were purchased from Taconic Farms (Hudson, NY). To determine viral spread of DOG4 in the CNS with and without TriGAS post-exposure prophylaxis (PET), groups of 10–15 mice were inoculated intranasally (i.n.) with 20 µl containing 10^5^ FFU of live DOG4 and 4 hrs later, either mock-treated (PBS) or administered with 10^7^ FFU TriGAS in the masseter muscle (i.m.). At various times later, mice were cardiac perfused, brain tissues were removed, and total RNA was isolated using the RNeasy minikit (Qiagen, Valencia, CA) according to the manufacturer's instructions. For quantification of RABV N protein RNA, reverse transcription (RT) and quantitative PCR (qPCR) were performed as described previously [10], [55]. Briefly, cDNA was synthesized by reverse transcription using the IScript cDNA synthesis kit (Bio-Rad). qPCR was performed on the iCycler iQ real-time detection system (Bio-Rad) using iQ Supermix (Bio-Rad) with gene specific primers and probe for RABV N of DOG4, whose sequences have been described previously [55]. RNA copy numbers for RABV N were normalized to the copy numbers of the housekeeping gene L13 in each sample as detailed elsewhere [56]. Statistical significance of the differences between groups was determined using the Mann-Whitney test.

### Metabolome analysis of biomarkers associated with rabies infection and post-infection viral clearance facilitated by TriGAS treatment

Two groups of 18 female C57BL/6 mice were infected i.n. with 10^5^ FFU of DOG4. Four hours post infection, one group was treated by intramuscular (i.m., masseter) injection of TriGAS vaccine in PBS, the other group mock-treated with PBS vehicle. An additional uninfected, untreated group served as a reference for metabolic profiling. Global biochemical profiles of 259 biochemicals were determined in whole brain tissue samples taken upon euthanasia at 4, 6 and 8 days post infection. Metabolic profiling performed at Metabolon, Inc. (Research Triangle Park, NC) combined three independent platforms: ultrahigh performance liquid chromatography/tandem mass spectrometry (UHLC/MS/MS2) optimized for basic species, UHLC/MS/MS2 optimized for acidic species, and gas chromatography/mass spectrometry (GC/MS). Metabolites were identified by automated comparison of the ion features in the experimental samples to a reference library of chemical standard entries developed at Metabolon. Following log transformation and imputation with minimum observed values for each compound, Welch's two-sample t-tests were used to identify biochemicals that differed significantly between 1) infected, mock-treated, 2) infected, vaccine treated and in 3) healthy non-infected, non-treated mice at respective time points.

### WT RABV infection and PET treatment with metyrapone, an 11-β hydroxylase inhibitor

To ensure metyrapone administration was not toxic in the absence of infection, 20 C57BL6 mice were administered 100 or 200 mg/kg intraperitoneally (i.p.) daily for 21 days. Mice displayed neither body weight loss nor overt clinical symptoms, with the exception of 200 mg/kg causing temporary sedation. The latter dose was not used in the subsequent experiments. Two additional groups of 20 female C57BL6 mice were infected i.n. with 10^4^ FFU of DOG4. The lower dose of DOG4, which consistently kills 90% of untreated animals, was chosen to better enable the detection of any therapeutic effect. Four hours later, mice were administered either metyrapone (100 mg/kg) or phosphate-buffered saline (PBS) i.p. each day for 21 days. Mice were observed daily for at least 28 days for clinical signs of rabies, and body weights and survival were recorded. Moribund animals were euthanized. Clinical signs were scored as follows: 1 = Disordered movement, piloerection; 2 = Hunched back, abnormal gait; 3 = Shaking/trembling, partial paralysis; 4 = Paralysis; 5 = Moribund/death.

### Measurement of serum corticosterone

Eight 10 week old female C57BL/6 mice were briefly anesthetized and bled using tail vein nick two days prior to i.n. infection with 10^4^ or 10^5^ FFU of DOG4 or mock infection and then after PBS or vaccine treatment (as described above) and on days 4, 6, 8 and 10 p.i. To control for natural diurnal fluctuations in levels of corticosterone, mice were bled between the hours of 9:00 am and 11:00 am each day. 25 µl serum from each animal was placed on ice and then spun at 2500× *g* at 4°C for 20 min. Plasma was stored at −80°C until assay. Corticosterone was measured by corticosterone EIA (Arbor Assays, Ann Arbor, MI) in duplicate according to manufacturer's instructions.

All experiments with mice were conducted in accordance with the Public Health Service Policy on Humane Care and Use of Laboratory Animals under protocols approved by the Institutional Animal Care and Use Committee of Thomas Jefferson University (Animal Welfare Assurance no. A3085-01).

## Supporting Information

Figure S1 **Principal component analysis reveals changes in brain metabolism when comparing infected, mock-treated and infected, TriGAS-treated mice.** Metabolites cluster together according to time and treatment with the exception of the day 8 samples, indicating an increasing number of statistically significant differences between infected, mock-treated and infected, TriGAS-treated animals as the infection progresses.
(TIF)

Figure S2 **WT RABV infects the adrenal glands late in infection.** Mice were infected i.n. with 10^5^ FFU of DOG4 RABV. Fifteen mice were euthanized and the number of DOG4 RABV N mRNA copies in both adrenal glands was quantified by qRT-PCR as described in Materials and Methods. The results are presented as the mean N mRNA copy numbers (+/− SE) per 1,000 copies of L13 mRNA.
(TIF)

Figure S3 **WT RABV results in hypoglycemia late in infection.** Groups of 10 C57BL/6 mice were bled 2 days prior to infection, infected with 10^5^ FFU of DOG4 RABV (closed bars) or mock-infected with PBS (open bars) and bled 8 days later. Plasma was separated and stored as described in Materials and Methods. Blood glucose levels were measured using Nipro Diagnostics True Track (Fort Lauderdale, FL) glucose meter, microchip #3614. Values represent group means attained from pooling 5 µl serum from each animal. Individual electronic strips were used for each group. Intrastrip variability was <5%, as determined by through calibration.
(TIF)

Table S1 **Time-dependent depiction and profile of brain metabolic changes that may distinguish between the progression and clearance of RABV infection.** The heat map shows metabolites differentially expressed in the brains of a) DOG4-infected, mock-treated (Mock Treatment); b) DOG4-infected, TriGAS-treated (Vaccine Treatment) and in c) healthy, uninfected and untreated (Uninfected Control) mice at 4, 6 and 8 days p.i.
(DOCX)

Table S2 **PET treatment with metyrapone has no effect on WT RABV replication in the CNS over the first 8 days of infection.**
(DOCX)

Table S3 **The metyrapone-treated mice that survived WT RABV infection develop significant serum RABV-specific neutralizing antibodies (VNA) titers.**
(DOCX)
