# Supplementary figures and images for: Limited Brain Metabolism Changes Differentiate between the Progression and Clearance of Rabies Virus
Source: PLoS One. 2014 Apr 24;9(4):e87180. doi: 10.1371/journal.pone.0087180 (PMC3998930; doi:10.1371/journal.pone.0087180)

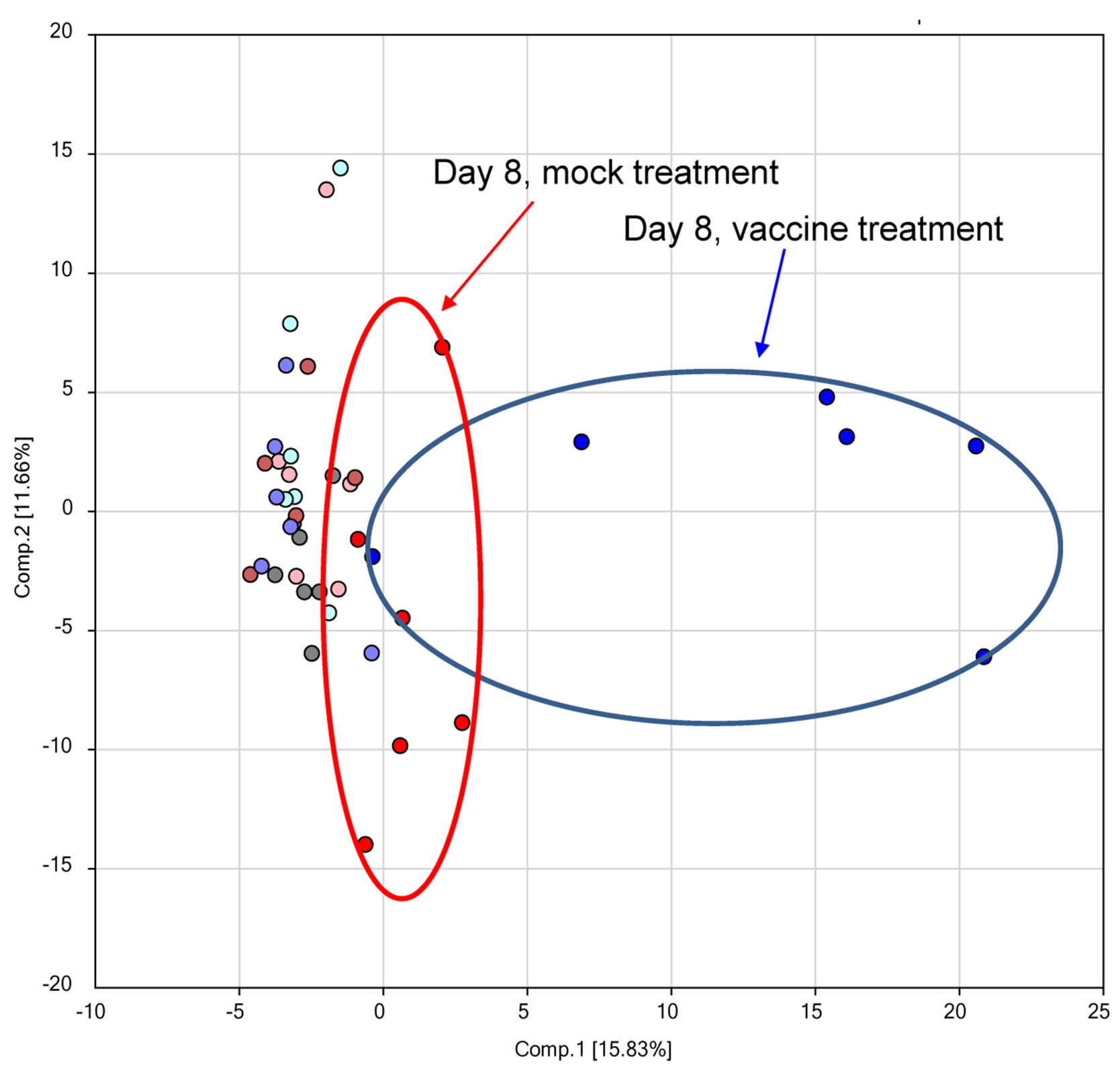

Supplement: Figure S1 — Principal component analysis reveals changes in brain metabolism when comparing infected, mock-treated and infected, TriGAS-treated mice. Metabolites cluster together according to time and treatment with the exception of the day 8 samples, indicating an increasing number of statistically significant differences between infected, mock-treated and infected, TriGAS-treated animals as the infection progresses. (TIF) [file pone.0087180.s001.tif]

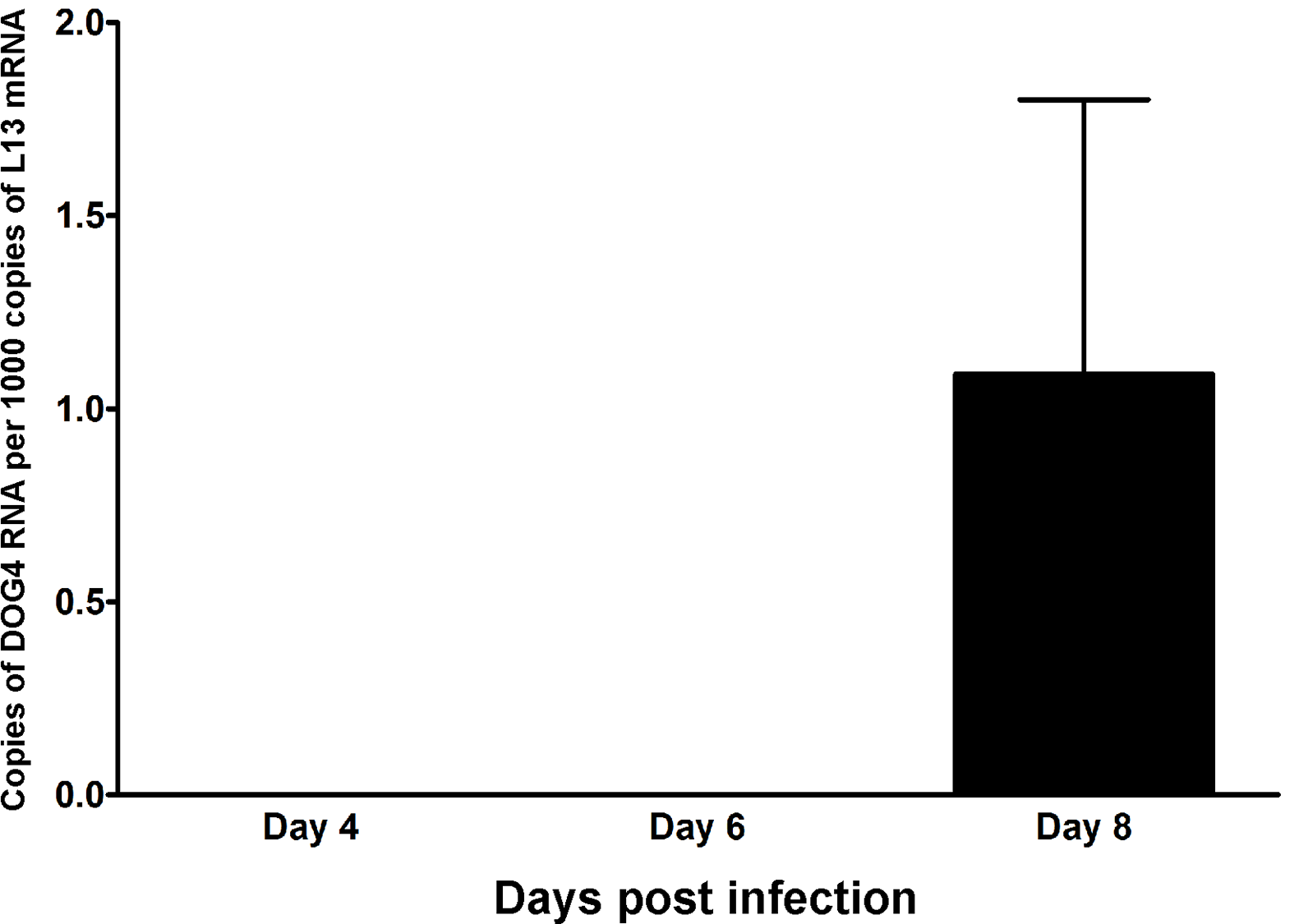

Supplement: Figure S2 — WT RABV infects the adrenal glands late in infection. Mice were infected i.n. with 105 FFU of DOG4 RABV. Fifteen mice were euthanized and the number of DOG4 RABV N mRNA copies in both adrenal glands was quantified by qRT-PCR as described in Materials and Methods. The results are presented as the mean N mRNA copy numbers (+/− SE) per 1,000 copies of L13 mRNA. (TIF) [file pone.0087180.s002.tif]

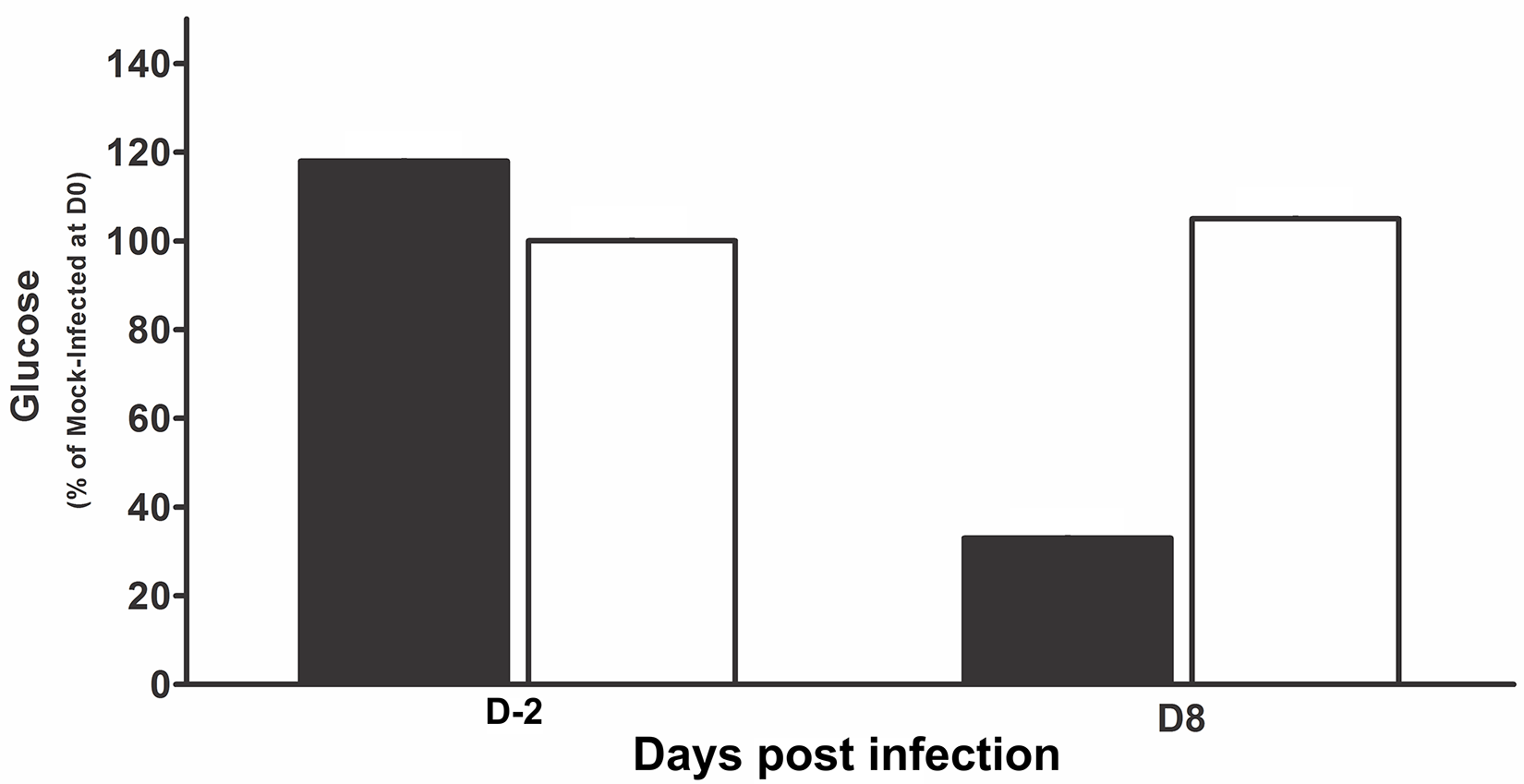

Supplement: Figure S3 — WT RABV results in hypoglycemia late in infection. Groups of 10 C57BL/6 mice were bled 2 days prior to infection, infected with 105 FFU of DOG4 RABV (closed bars) or mock-infected with PBS (open bars) and bled 8 days later. Plasma was separated and stored as described in Materials and Methods. Blood glucose levels were measured using Nipro Diagnostics True Track (Fort Lauderdale, FL) glucose meter, microchip #3614. Values represent group means attained from pooling 5 µl serum from each animal. Individual electronic strips were used for each group. Intrastrip variability was <5%, as determined by through calibration. (TIF) [file pone.0087180.s003.tif]
