# Supplementary material for: Limited Brain Metabolism Changes Differentiate between the Progression and Clearance of Rabies Virus
Source: PLoS One. 2014 Apr 24;9(4):e87180. doi: 10.1371/journal.pone.0087180 (PMC3998930; doi:10.1371/journal.pone.0087180)
Supplement: Table S1 — Time-dependent depiction and profile of brain metabolic changes that may distinguish between the progression and clearance of RABV infection. The heat map shows metabolites differentially expressed in the brains of a) DOG4-infected, mock-treated (Mock Treatment); b) DOG4-infected, TriGAS-treated (Vaccine Treatment) and in c) healthy, uninfected and untreated (Uninfected Control) mice at 4, 6 and 8 days p.i. (DOCX) [file pone.0087180.s004.docx]

**Supporting Information Tables**

**Table S1. Time-dependent depiction and profile of brain metabolic changes that may distinguish between the progression and clearance of RABV infection.** The heat map shows metabolites differentially expressed in the brains of a) DOG4-infected, mock-treated (Mock Treatment); b) DOG4-infected, TriGAS-treated (Vaccine Treatment) and in c) healthy, uninfected and untreated (Uninfected Control) mice at 4, 6 and 8 days p.i.

| **Red = Upregulated >1.5** | **Comparative levels** | | | | | | | | |
| --- | --- | --- | --- | --- | --- | --- | --- | --- | --- |
| **Green = Downregulated <0.75** | **Welch's Two-Sample t-Test** | | | | | | | | |
|  | **Mock Treatment / Uninfected Control** | | | **Vaccine Treatment / Uninfected Control** | | | **Vaccine Treatment / Mock Treatment** | | |
| **BIOCHEMICAL NAME** | **Day 4** | **Day 6** | **Day 8** | **Day 4** | **Day 6** | **Day 8** | **Day 4** | **Day 6** | **Day 8** |
|  |  |  |  |  |  |  |  |  |  |
| **glycine** | **1.16** | **0.89** | **1.00** | **1.05** | **0.92** | **1.35** | **0.91** | **1.03** | **1.35** |
| **dimethylglycine** | **1.11** | **0.95** | **1.10** | **0.90** | **1.05** | **1.75** | **0.81** | **1.11** | **1.59** |
| **N-acetylglycine** | **1.34** | **1.22** | **1.43** | **0.89** | **0.82** | **2.91** | **0.67** | **0.67** | **2.04** |
| **serine** | **1.04** | **1.06** | **0.87** | **1.06** | **1.05** | **1.18** | **1.02** | **1.00** | **1.36** |
| **homoserine** | **0.88** | **0.97** | **0.68** | **0.98** | **0.81** | **0.91** | **1.11** | **0.84** | **1.34** |
| **threonine** | **1.14** | **0.95** | **1.05** | **1.13** | **0.88** | **1.68** | **0.99** | **0.92** | **1.61** |
| **allo-threonine** | **1.20** | **1.08** | **1.03** | **1.14** | **0.99** | **1.51** | **0.95** | **0.91** | **1.46** |
| **betaine** | **0.98** | **0.98** | **1.05** | **0.86** | **1.04** | **3.30** | **0.88** | **1.06** | **3.13** |
| **aspartate** | **0.96** | **1.07** | **0.99** | **0.98** | **1.00** | **1.07** | **1.02** | **0.93** | **1.08** |
| **beta-alanine** | **1.15** | **1.16** | **1.19** | **1.09** | **1.40** | **1.24** | **0.95** | **1.20** | **1.05** |
| **alanine** | **0.92** | **1.00** | **0.96** | **0.99** | **0.97** | **1.03** | **1.07** | **0.98** | **1.07** |
| **N-acetylaspartate (NAA)** | **1.04** | **1.04** | **0.94** | **1.06** | **0.99** | **0.90** | **1.02** | **0.96** | **0.96** |
| **glutamate** | **1.03** | **1.00** | **0.97** | **1.04** | **1.02** | **1.00** | **1.01** | **1.01** | **1.02** |
| **glutamate, gamma-methyl ester** | **1.21** | **1.24** | **1.21** | **1.46** | **1.35** | **1.74** | **1.21** | **1.09** | **1.44** |
| **glutamine** | **1.07** | **0.90** | **0.98** | **1.09** | **0.92** | **1.07** | **1.02** | **1.02** | **1.10** |
| **pyroglutamine*** | **1.14** | **1.05** | **0.74** | **1.15** | **1.23** | **0.87** | **1.01** | **1.17** | **1.18** |
| **gamma-aminobutyrate (GABA)** | **1.09** | **1.07** | **1.16** | **1.09** | **1.21** | **1.09** | **1.00** | **1.13** | **0.94** |
| **N-acetylglutamate** | **1.03** | **1.00** | **1.21** | **1.08** | **0.98** | **1.12** | **1.05** | **0.98** | **0.92** |
| **N-acetyl-aspartyl-glutamate (NAAG)** | **1.17** | **0.97** | **1.17** | **1.08** | **0.95** | **1.05** | **0.92** | **0.99** | **0.90** |
| **histidine** | **1.10** | **0.89** | **0.89** | **1.07** | **0.95** | **1.23** | **0.97** | **1.07** | **1.37** |
| **trans-urocanate** | **1.50** | **1.18** | **1.23** | **1.17** | **1.04** | **0.69** | **0.78** | **0.88** | **0.56** |
| **1-methylimidazoleacetate** | **0.91** | **1.00** | **1.39** | **0.90** | **0.81** | **0.89** | **0.99** | **0.81** | **0.64** |
| **lysine** | **1.02** | **0.93** | **0.98** | **1.09** | **0.98** | **1.55** | **1.07** | **1.05** | **1.59** |
| **2-aminoadipate** | **0.89** | **1.22** | **1.17** | **1.00** | **1.07** | **2.55** | **1.12** | **0.88** | **2.18** |
| **pipecolate** | **1.10** | **1.16** | **0.79** | **1.03** | **1.20** | **1.70** | **0.93** | **1.04** | **2.16** |
| **glutaroyl carnitine** | **1.21** | **1.17** | **1.00** | **1.23** | **1.14** | **1.14** | **1.01** | **0.97** | **1.15** |
| **phenylalanine** | **1.08** | **0.87** | **1.35** | **1.09** | **0.96** | **1.23** | **1.01** | **1.10** | **0.91** |
| **tyrosine** | **0.90** | **0.95** | **0.61** | **1.00** | **0.98** | **0.97** | **1.11** | **1.03** | **1.59** |
| **3-(4-hydroxyphenyl)lactate** | **0.90** | **0.94** | **0.75** | **0.97** | **0.98** | **1.09** | **1.08** | **1.04** | **1.46** |
| **dopamine** | **1.16** | **1.14** | **1.26** | **1.13** | **1.44** | **1.12** | **0.97** | **1.26** | **0.89** |
| **N-acetylphenylalanine** | **1.21** | **1.00** | **2.46** | **1.15** | **1.06** | **2.67** | **0.95** | **1.06** | **1.08** |
| **phenol sulfate** | **0.72** | **0.89** | **0.99** | **0.75** | **1.07** | **1.12** | **1.04** | **1.20** | **1.14** |
| **kynurenine** | **1.13** | **1.39** | **1.77** | **1.03** | **1.11** | **4.11** | **0.91** | **0.80** | **2.32** |
| **tryptophan** | **1.00** | **0.81** | **1.08** | **0.94** | **0.86** | **1.09** | **0.94** | **1.05** | **1.01** |
| **serotonin (5HT)** | **1.03** | **0.93** | **0.90** | **0.85** | **0.93** | **0.91** | **0.83** | **1.00** | **1.01** |
| **C-glycosyltryptophan*** | **1.09** | **1.05** | **0.93** | **1.14** | **1.05** | **0.92** | **1.04** | **1.00** | **0.98** |
| **5-hydroxyindoleacetate** | **0.89** | **0.79** | **2.00** | **0.88** | **0.85** | **1.54** | **0.99** | **1.08** | **0.77** |
| **levulinate (4-oxovalerate)** | **1.08** | **0.88** | **0.98** | **0.90** | **1.07** | **1.08** | **0.84** | **1.21** | **1.11** |
| **beta-hydroxyisovalerate** | **0.94** | **0.90** | **1.07** | **1.26** | **0.92** | **1.09** | **1.34** | **1.03** | **1.02** |
| **isoleucine** | **1.04** | **1.04** | **0.96** | **1.15** | **0.91** | **1.03** | **1.10** | **0.87** | **1.06** |
| **leucine** | **1.10** | **1.02** | **0.94** | **1.14** | **0.89** | **1.02** | **1.04** | **0.88** | **1.09** |
| **valine** | **1.05** | **1.08** | **1.11** | **1.14** | **1.00** | **1.17** | **1.09** | **0.93** | **1.05** |
| **alpha-hydroxyisovalerate** | **0.92** | **1.00** | **2.10** | **1.34** | **0.87** | **1.17** | **1.46** | **0.87** | **0.56** |
| **isobutyrylcarnitine** | **1.52** | **1.18** | **1.66** | **1.44** | **1.28** | **3.49** | **0.95** | **1.09** | **2.10** |
| **2-methylbutyroylcarnitine** | **1.30** | **1.02** | **1.38** | **1.21** | **1.05** | **3.52** | **0.93** | **1.04** | **2.55** |
| **isovalerylcarnitine** | **1.65** | **1.17** | **1.44** | **1.32** | **1.22** | **4.49** | **0.79** | **1.04** | **3.11** |
| **hydroxyisovaleroyl carnitine** | **1.10** | **1.00** | **1.07** | **1.08** | **1.04** | **1.38** | **0.98** | **1.04** | **1.29** |
| **cysteine** | **1.64** | **1.92** | **2.01** | **1.17** | **1.49** | **1.50** | **0.71** | **0.77** | **0.75** |
| **N-formylmethionine** | **1.11** | **1.09** | **1.29** | **1.25** | **1.01** | **1.24** | **1.12** | **0.92** | **0.96** |
| **hypotaurine** | **1.34** | **1.14** | **1.29** | **1.31** | **1.28** | **3.32** | **0.98** | **1.13** | **2.57** |
| **taurine** | **1.05** | **1.21** | **0.90** | **1.17** | **1.20** | **1.15** | **1.11** | **0.99** | **1.27** |
| **S-adenosylhomocysteine (SAH)** | **1.08** | **0.93** | **0.94** | **1.00** | **1.03** | **0.97** | **0.93** | **1.11** | **1.04** |
| **methionine** | **0.98** | **0.91** | **0.66** | **0.98** | **0.86** | **0.98** | **1.00** | **0.94** | **1.49** |
| **N-acetylmethionine** | **0.91** | **0.95** | **0.93** | **0.97** | **0.87** | **1.05** | **1.07** | **0.92** | **1.13** |
| **2-hydroxybutyrate (AHB)** | **0.87** | **0.89** | **3.29** | **1.00** | **0.79** | **1.38** | **1.14** | **0.89** | **0.42** |
| **arginine** | **1.02** | **0.96** | **0.91** | **1.09** | **0.95** | **1.18** | **1.07** | **0.99** | **1.30** |
| **urea** | **1.16** | **0.96** | **1.22** | **1.12** | **0.91** | **0.80** | **0.96** | **0.95** | **0.65** |
| **proline** | **1.08** | **1.01** | **1.09** | **1.07** | **1.01** | **1.22** | **0.99** | **1.00** | **1.12** |
| **citrulline** | **0.93** | **0.84** | **0.66** | **0.96** | **0.78** | **0.85** | **1.04** | **0.93** | **1.28** |
| **N-acetylornithine** | **1.05** | **1.15** | **0.32** | **1.06** | **1.07** | **1.09** | **1.01** | **0.93** | **3.41** |
| **trans-4-hydroxyproline** | **1.18** | **1.03** | **0.40** | **1.18** | **0.69** | **1.59** | **1.00** | **0.66** | **3.94** |
| **creatine** | **1.01** | **0.96** | **0.94** | **1.02** | **1.05** | **0.96** | **1.01** | **1.09** | **1.02** |
| **creatinine** | **1.00** | **0.97** | **0.90** | **1.07** | **0.98** | **0.97** | **1.06** | **1.02** | **1.08** |
| **2-aminobutyrate** | **0.73** | **0.93** | **2.25** | **1.04** | **0.86** | **1.82** | **1.43** | **0.92** | **0.81** |
| **5-methylthioadenosine (MTA)** | **1.00** | **0.77** | **0.91** | **0.83** | **0.89** | **0.95** | **0.83** | **1.17** | **1.05** |
| **putrescine** | **1.15** | **0.89** | **5.03** | **1.01** | **1.30** | **6.38** | **0.88** | **1.46** | **1.27** |
| **spermidine** | **1.16** | **0.75** | **0.91** | **0.85** | **0.90** | **0.97** | **0.73** | **1.20** | **1.07** |
| **4-guanidinobutanoate** | **1.03** | **0.89** | **0.83** | **1.10** | **1.00** | **1.10** | **1.07** | **1.13** | **1.32** |
| **glutathione, reduced (GSH)** | **1.33** | **1.66** | **1.42** | **1.53** | **1.31** | **1.30** | **1.15** | **0.79** | **0.91** |
| **S-methylglutathione** | **1.11** | **1.02** | **0.88** | **0.83** | **0.95** | **0.96** | **0.75** | **0.92** | **1.10** |
| **5-oxoproline** | **1.02** | **0.77** | **0.80** | **1.01** | **0.91** | **0.97** | **1.00** | **1.18** | **1.22** |
| **glutathione, oxidized (GSSG)** | **1.02** | **1.00** | **0.89** | **1.02** | **1.07** | **1.05** | **1.00** | **1.07** | **1.18** |
| **ophthalmate** | **0.71** | **0.88** | **2.21** | **0.84** | **0.83** | **1.57** | **1.19** | **0.94** | **0.71** |
| **glycylisoleucine** | **1.38** | **1.34** | **1.46** | **0.83** | **0.91** | **1.03** | **0.60** | **0.68** | **0.71** |
| **glycylleucine** | **1.78** | **1.35** | **1.64** | **1.21** | **1.06** | **1.55** | **0.68** | **0.79** | **0.95** |
| **cysteinylglycine** | **1.16** | **1.24** | **0.99** | **1.19** | **1.61** | **1.32** | **1.03** | **1.29** | **1.33** |
| **leucylglycine** | **1.23** | **1.05** | **1.17** | **0.69** | **0.75** | **0.90** | **0.56** | **0.72** | **0.77** |
| **carnosine** | **1.14** | **1.00** | **1.09** | **1.12** | **0.99** | **0.99** | **0.99** | **0.99** | **0.91** |
| **homocarnosine** | **1.16** | **0.98** | **1.01** | **1.05** | **1.03** | **0.95** | **0.90** | **1.05** | **0.94** |
| **anserine** | **1.37** | **1.15** | **1.25** | **1.33** | **1.22** | **1.50** | **0.97** | **1.06** | **1.20** |
| **gamma-glutamylvaline** | **0.88** | **0.82** | **0.88** | **1.06** | **0.91** | **1.04** | **1.20** | **1.11** | **1.18** |
| **gamma-glutamylleucine** | **0.95** | **0.90** | **0.94** | **1.11** | **0.95** | **1.17** | **1.16** | **1.05** | **1.25** |
| **gamma-glutamylglutamate** | **1.04** | **0.86** | **0.90** | **0.96** | **0.82** | **0.68** | **0.93** | **0.96** | **0.76** |
| **gamma-glutamylglutamine** | **0.99** | **0.72** | **0.77** | **1.01** | **0.77** | **0.74** | **1.02** | **1.06** | **0.97** |
| **erythronate*** | **0.99** | **0.93** | **1.02** | **0.97** | **0.90** | **0.62** | **0.98** | **0.97** | **0.61** |
| **1N-acetylneuraminate** | **1.36** | **1.34** | **1.14** | **1.28** | **1.10** | **0.97** | **0.95** | **0.82** | **0.85** |
| **fructose** | **1.08** | **1.20** | **1.47** | **1.04** | **1.07** | **1.47** | **0.96** | **0.89** | **1.00** |
| **6'-sialyllactose** | **1.12** | **1.20** | **1.28** | **1.26** | **1.05** | **1.13** | **1.13** | **0.88** | **0.88** |
| **mannitol** | **1.22** | **1.31** | **1.31** | **1.18** | **1.20** | **0.89** | **0.96** | **0.92** | **0.68** |
| **mannose-6-phosphate** | **1.37** | **1.81** | **1.91** | **1.58** | **1.25** | **2.10** | **1.15** | **0.69** | **1.10** |
| **sorbitol** | **1.31** | **1.25** | **1.45** | **1.30** | **1.20** | **1.24** | **0.99** | **0.96** | **0.86** |
| **1,5-anhydroglucitol (1,5-AG)** | **1.65** | **1.47** | **1.13** | **1.47** | **1.44** | **1.50** | **0.89** | **0.98** | **1.32** |
| **glycerate** | **0.99** | **1.01** | **0.97** | **0.95** | **1.03** | **1.24** | **0.96** | **1.02** | **1.27** |
| **glucose-6-phosphate (G6P)** | **0.89** | **1.32** | **1.78** | **0.90** | **0.85** | **2.54** | **1.01** | **0.64** | **1.43** |
| **glucose** | **1.22** | **1.82** | **1.47** | **1.14** | **1.11** | **1.49** | **0.94** | **0.61** | **1.01** |
| **fructose-6-phosphate** | **1.17** | **1.39** | **1.38** | **1.28** | **1.13** | **2.95** | **1.10** | **0.81** | **2.14** |
| **Isobar: fructose 1,6-diphosphate, glucose 1,6-diphosphate, myo-inositol 1,4 or 1,3-diphosphate** | **0.97** | **1.17** | **1.24** | **0.98** | **1.23** | **2.41** | **1.02** | **1.05** | **1.94** |
| **3-phosphoglycerate** | **1.30** | **1.40** | **1.25** | **1.45** | **1.12** | **2.50** | **1.11** | **0.80** | **1.99** |
| **lactate** | **0.95** | **0.99** | **1.02** | **0.97** | **0.97** | **1.02** | **1.03** | **0.98** | **1.00** |
| **6-phosphogluconate** | **0.91** | **0.95** | **0.84** | **0.99** | **0.93** | **0.90** | **1.10** | **0.98** | **1.07** |
| **arabitol** | **1.01** | **0.96** | **0.65** | **0.89** | **0.84** | **0.47** | **0.88** | **0.87** | **0.72** |
| **ribitol** | **0.93** | **0.94** | **0.81** | **0.97** | **0.93** | **0.79** | **1.05** | **0.98** | **0.98** |
| **arabinose** | **1.30** | **1.94** | **2.11** | **1.49** | **1.14** | **0.70** | **1.14** | **0.59** | **0.33** |
| **xylonate** | **1.33** | **0.96** | **0.68** | **1.56** | **1.17** | **1.05** | **1.17** | **1.21** | **1.53** |
| **citrate** | **1.10** | **1.21** | **1.01** | **1.17** | **1.11** | **1.35** | **1.07** | **0.92** | **1.34** |
| **succinate** | **1.06** | **1.01** | **0.98** | **1.09** | **1.00** | **1.13** | **1.03** | **0.98** | **1.14** |
| **succinylcarnitine** | **1.10** | **0.99** | **1.05** | **1.07** | **0.94** | **1.40** | **0.98** | **0.96** | **1.34** |
| **fumarate** | **1.00** | **0.93** | **0.90** | **0.97** | **1.00** | **0.88** | **0.97** | **1.07** | **0.98** |
| **malate** | **0.99** | **0.97** | **1.04** | **0.98** | **0.94** | **0.96** | **0.99** | **0.97** | **0.93** |
| **acetylphosphate** | **1.00** | **1.05** | **1.05** | **1.01** | **1.08** | **1.02** | **1.01** | **1.03** | **0.97** |
| **phosphate** | **1.05** | **1.03** | **1.01** | **1.08** | **1.02** | **0.98** | **1.03** | **0.98** | **0.98** |
| **pyrophosphate (PPi)** | **0.96** | **1.03** | **0.92** | **0.85** | **0.97** | **1.00** | **0.89** | **0.93** | **1.09** |
| **linoleate (18:2n6)** | **0.85** | **0.83** | **1.10** | **0.91** | **0.84** | **0.96** | **1.07** | **1.01** | **0.87** |
| **linolenate [alpha or gamma; (18:3n3 or 6)]** | **1.14** | **1.13** | **1.12** | **1.74** | **1.03** | **1.19** | **1.53** | **0.91** | **1.06** |
| **dihomo-linolenate (20:3n3 or n6)** | **0.81** | **0.85** | **0.82** | **1.01** | **0.84** | **0.74** | **1.25** | **0.99** | **0.90** |
| **docosapentaenoate (n3 DPA; 22:5n3)** | **1.05** | **1.11** | **1.04** | **1.41** | **1.20** | **0.93** | **1.34** | **1.09** | **0.89** |
| **docosapentaenoate (n6 DPA; 22:5n6)** | **1.17** | **1.36** | **1.10** | **1.70** | **1.24** | **1.16** | **1.46** | **0.92** | **1.05** |
| **docosahexaenoate (DHA; 22:6n3)** | **1.03** | **0.95** | **1.01** | **1.14** | **1.06** | **0.89** | **1.11** | **1.12** | **0.89** |
| **caproate (6:0)** | **0.96** | **0.91** | **1.06** | **1.01** | **1.11** | **1.05** | **1.05** | **1.22** | **0.99** |
| **caprylate (8:0)** | **0.73** | **0.65** | **0.81** | **0.76** | **0.73** | **0.88** | **1.04** | **1.13** | **1.09** |
| **pelargonate (9:0)** | **0.81** | **0.67** | **0.80** | **0.82** | **0.77** | **0.73** | **1.01** | **1.14** | **0.91** |
| **caprate (10:0)** | **0.83** | **0.67** | **0.78** | **0.81** | **0.83** | **0.84** | **0.98** | **1.24** | **1.09** |
| **undecanoate (11:0)** | **0.64** | **0.72** | **0.84** | **0.77** | **0.79** | **0.88** | **1.20** | **1.10** | **1.04** |
| **laurate (12:0)** | **0.90** | **0.83** | **0.94** | **0.89** | **0.88** | **0.90** | **0.99** | **1.06** | **0.95** |
| **myristate (14:0)** | **0.98** | **1.04** | **0.99** | **1.00** | **1.06** | **1.00** | **1.02** | **1.02** | **1.02** |
| **myristoleate (14:1n5)** | **0.89** | **0.91** | **0.92** | **1.01** | **0.89** | **1.17** | **1.14** | **0.98** | **1.27** |
| **palmitate (16:0)** | **0.99** | **1.09** | **1.02** | **1.08** | **1.02** | **0.90** | **1.09** | **0.94** | **0.88** |
| **palmitoleate (16:1n7)** | **0.96** | **1.01** | **0.92** | **1.05** | **0.94** | **0.84** | **1.09** | **0.93** | **0.92** |
| **margarate (17:0)** | **0.97** | **1.13** | **0.99** | **0.94** | **1.00** | **0.90** | **0.97** | **0.88** | **0.91** |
| **stearate (18:0)** | **1.07** | **1.32** | **1.18** | **1.18** | **1.13** | **1.00** | **1.11** | **0.85** | **0.85** |
| **oleate (18:1n9)** | **0.89** | **0.82** | **0.95** | **0.91** | **0.89** | **0.76** | **1.02** | **1.09** | **0.80** |
| **cis-vaccenate (18:1n7)** | **0.95** | **0.82** | **0.89** | **0.94** | **0.89** | **0.72** | **0.99** | **1.09** | **0.80** |
| **10-nonadecenoate (19:1n9)** | **0.92** | **1.04** | **0.98** | **1.07** | **1.03** | **0.86** | **1.16** | **0.99** | **0.88** |
| **eicosenoate (20:1n9 or 11)** | **1.04** | **1.02** | **1.13** | **1.19** | **0.98** | **0.90** | **1.15** | **0.96** | **0.80** |
| **mead acid (20:3n9)** | **1.04** | **0.97** | **0.86** | **1.15** | **0.95** | **0.60** | **1.11** | **0.97** | **0.69** |
| **arachidonate (20:4n6)** | **0.97** | **1.02** | **0.95** | **1.03** | **1.08** | **0.87** | **1.06** | **1.05** | **0.92** |
| **adrenate (22:4n6)** | **0.95** | **0.76** | **0.74** | **0.90** | **0.83** | **0.79** | **0.95** | **1.10** | **1.07** |
| **2-hydroxyglutarate** | **0.95** | **0.98** | **1.15** | **0.95** | **0.94** | **1.00** | **1.00** | **0.96** | **0.88** |
| **13-methylmyristic acid** | **1.08** | **1.14** | **1.11** | **1.19** | **0.98** | **1.04** | **1.10** | **0.86** | **0.93** |
| **propionylcarnitine** | **0.95** | **0.96** | **1.03** | **1.00** | **1.01** | **1.40** | **1.06** | **1.04** | **1.36** |
| **butyrylcarnitine** | **1.07** | **0.93** | **0.86** | **1.06** | **0.96** | **1.75** | **1.00** | **1.03** | **2.03** |
| **valerylcarnitine** | **0.85** | **0.87** | **0.95** | **0.90** | **0.88** | **1.06** | **1.05** | **1.01** | **1.12** |
| **deoxycarnitine** | **0.95** | **0.99** | **1.00** | **1.00** | **1.01** | **1.13** | **1.05** | **1.02** | **1.13** |
| **carnitine** | **1.08** | **1.00** | **1.04** | **1.04** | **1.01** | **1.70** | **0.96** | **1.01** | **1.64** |
| **3-dehydrocarnitine*** | **1.18** | **0.98** | **1.06** | **1.01** | **1.14** | **2.02** | **0.86** | **1.17** | **1.91** |
| **acetylcarnitine** | **1.03** | **1.05** | **0.98** | **1.02** | **1.03** | **1.62** | **0.99** | **0.98** | **1.66** |
| **hexanoylcarnitine** | **1.26** | **1.31** | **1.28** | **1.18** | **1.12** | **2.38** | **0.93** | **0.85** | **1.85** |
| **octanoylcarnitine** | **0.95** | **0.99** | **1.42** | **1.08** | **0.90** | **2.66** | **1.13** | **0.90** | **1.88** |
| **laurylcarnitine** | **0.78** | **0.74** | **1.04** | **0.77** | **0.91** | **2.12** | **0.98** | **1.23** | **2.03** |
| **palmitoylcarnitine** | **0.67** | **0.75** | **0.98** | **0.81** | **0.86** | **1.77** | **1.22** | **1.14** | **1.80** |
| **stearoylcarnitine** | **0.79** | **0.84** | **1.06** | **1.07** | **0.93** | **2.12** | **1.35** | **1.11** | **2.01** |
| **oleoylcarnitine** | **0.66** | **0.71** | **0.78** | **0.78** | **0.80** | **1.58** | **1.18** | **1.13** | **2.02** |
| **1-octadecanol** | **0.77** | **0.87** | **0.86** | **0.87** | **0.73** | **0.74** | **1.12** | **0.83** | **0.87** |
| **choline phosphate** | **0.94** | **1.01** | **1.02** | **0.97** | **0.97** | **1.20** | **1.03** | **0.96** | **1.17** |
| **ethanolamine** | **1.15** | **1.10** | **0.84** | **1.33** | **0.92** | **0.75** | **1.15** | **0.84** | **0.90** |
| **phosphoethanolamine** | **1.06** | **1.10** | **1.18** | **1.00** | **0.99** | **1.35** | **0.94** | **0.90** | **1.14** |
| **glycerol** | **1.05** | **1.04** | **0.97** | **1.11** | **1.04** | **0.99** | **1.05** | **1.00** | **1.01** |
| **glycerol 3-phosphate (G3P)** | **0.95** | **0.94** | **0.68** | **0.99** | **0.94** | **0.97** | **1.04** | **1.01** | **1.44** |
| **glycerophosphorylcholine (GPC)** | **0.90** | **0.99** | **0.59** | **0.88** | **1.02** | **0.76** | **0.99** | **1.03** | **1.30** |
| **cytidine-5'-diphosphoethanolamine** | **1.00** | **1.01** | **1.08** | **0.99** | **0.93** | **0.94** | **0.99** | **0.92** | **0.87** |
| **myo-inositol** | **1.05** | **1.00** | **0.94** | **1.04** | **1.01** | **0.90** | **0.98** | **1.01** | **0.95** |
| **chiro-inositol** | **1.00** | **0.95** | **0.64** | **0.87** | **0.79** | **1.27** | **0.88** | **0.84** | **1.99** |
| **inositol 1-phosphate (I1P)** | **1.03** | **1.25** | **0.97** | **0.91** | **1.00** | **0.94** | **0.89** | **0.80** | **0.96** |
| **scyllo-inositol** | **0.94** | **0.92** | **0.84** | **0.92** | **0.96** | **0.83** | **0.97** | **1.05** | **1.00** |
| **3-hydroxybutyrate (BHBA)** | **0.99** | **0.94** | **2.23** | **1.04** | **0.94** | **1.36** | **1.06** | **0.99** | **0.61** |
| **1,2-propanediol** | **1.07** | **1.00** | **0.93** | **1.17** | **1.01** | **0.90** | **1.09** | **1.02** | **0.96** |
| **1-palmitoylglycerophosphoethanolamine** | **1.03** | **1.00** | **1.11** | **0.91** | **1.05** | **1.36** | **0.88** | **1.06** | **1.22** |
| **2-palmitoylglycerophosphoethanolamine*** | **1.78** | **1.04** | **3.32** | **1.20** | **1.07** | **0.75** | **0.68** | **1.03** | **0.22** |
| **2-palmitoleoylglycerophosphoethanolamine*** | **0.94** | **0.66** | **1.10** | **0.96** | **0.69** | **0.87** | **1.01** | **1.04** | **0.79** |
| **1-stearoylglycerophosphoethanolamine** | **0.77** | **1.00** | **1.32** | **0.66** | **1.00** | **0.90** | **0.85** | **1.00** | **0.68** |
| **1-oleoylglycerophosphoethanolamine** | **0.99** | **0.80** | **1.11** | **0.75** | **1.02** | **0.96** | **0.76** | **1.27** | **0.87** |
| **2-oleoylglycerophosphoethanolamine*** | **0.95** | **0.95** | **1.19** | **0.96** | **1.03** | **1.14** | **1.01** | **1.09** | **0.96** |
| **1-arachidonoylglycerophosphoethanolamine*** | **0.96** | **0.84** | **0.92** | **0.81** | **1.05** | **0.87** | **0.84** | **1.25** | **0.95** |
| **2-arachidonoylglycerophosphoethanolamine*** | **0.86** | **0.78** | **1.01** | **0.79** | **0.74** | **0.84** | **0.92** | **0.95** | **0.83** |
| **2-docosapentaenoylglycerophosphoethanolamine*** | **1.10** | **0.90** | **1.06** | **0.65** | **0.63** | **0.69** | **0.60** | **0.70** | **0.65** |
| **2-docosahexaenoylglycerophosphoethanolamine*** | **0.82** | **0.80** | **0.99** | **0.80** | **0.76** | **0.86** | **0.98** | **0.95** | **0.87** |
| **1-myristoylglycerophosphocholine** | **0.92** | **0.93** | **0.94** | **0.85** | **0.87** | **0.96** | **0.92** | **0.94** | **1.02** |
| **2-myristoylglycerophosphocholine*** | **1.01** | **1.01** | **1.11** | **0.99** | **0.85** | **0.77** | **0.98** | **0.84** | **0.70** |
| **1-palmitoylglycerophosphocholine** | **0.71** | **0.77** | **0.98** | **0.96** | **0.79** | **0.75** | **1.35** | **1.04** | **0.76** |
| **2-palmitoylglycerophosphocholine*** | **0.85** | **0.70** | **0.98** | **0.93** | **0.71** | **0.86** | **1.09** | **1.00** | **0.88** |
| **1-palmitoleoylglycerophosphocholine*** | **1.04** | **0.82** | **0.99** | **0.71** | **0.72** | **0.77** | **0.69** | **0.87** | **0.77** |
| **2-palmitoleoylglycerophosphocholine*** | **1.04** | **0.79** | **1.23** | **0.98** | **0.87** | **0.94** | **0.94** | **1.10** | **0.76** |
| **1-stearoylglycerophosphocholine** | **0.70** | **0.59** | **0.78** | **0.86** | **0.85** | **0.83** | **1.23** | **1.45** | **1.07** |
| **2-stearoylglycerophosphocholine*** | **0.64** | **0.51** | **0.81** | **0.64** | **0.50** | **0.59** | **1.00** | **0.98** | **0.73** |
| **1-oleoylglycerophosphocholine** | **0.71** | **0.76** | **0.95** | **0.91** | **0.79** | **0.67** | **1.27** | **1.04** | **0.71** |
| **2-oleoylglycerophosphocholine*** | **0.75** | **0.71** | **1.06** | **1.10** | **0.70** | **0.73** | **1.46** | **0.99** | **0.68** |
| **1-linoleoylglycerophosphocholine** | **1.07** | **1.12** | **0.99** | **0.72** | **0.68** | **0.96** | **0.68** | **0.61** | **0.97** |
| **2-linoleoylglycerophosphocholine*** | **1.01** | **1.07** | **1.13** | **0.73** | **0.70** | **1.07** | **0.73** | **0.65** | **0.95** |
| **1-arachidonoylglycerophosphocholine*** | **1.02** | **0.91** | **0.85** | **0.53** | **0.68** | **0.97** | **0.52** | **0.75** | **1.14** |
| **2-arachidonoylglycerophosphocholine*** | **0.88** | **0.79** | **1.10** | **0.79** | **0.73** | **0.93** | **0.90** | **0.92** | **0.85** |
| **1-docosahexaenoylglycerophosphocholine*** | **1.00** | **0.81** | **0.97** | **0.63** | **0.77** | **0.90** | **0.63** | **0.95** | **0.92** |
| **2-docosahexaenoylglycerophosphocholine*** | **0.84** | **0.83** | **1.14** | **0.88** | **0.79** | **0.95** | **1.05** | **0.95** | **0.84** |
| **1-palmitoylglycerophosphoinositol*** | **0.98** | **0.92** | **1.16** | **1.07** | **0.95** | **0.74** | **1.09** | **1.03** | **0.64** |
| **1-stearoylglycerophosphoinositol** | **1.11** | **1.11** | **1.15** | **1.20** | **1.04** | **0.94** | **1.08** | **0.94** | **0.81** |
| **1-oleoylglycerophosphoinositol*** | **0.87** | **0.81** | **0.91** | **0.86** | **0.95** | **0.68** | **0.99** | **1.17** | **0.75** |
| **1-arachidonoylglycerophosphoinositol*** | **0.98** | **0.88** | **1.08** | **1.13** | **1.00** | **0.80** | **1.15** | **1.13** | **0.74** |
| **1-oleoylglycerophosphoserine** | **1.07** | **0.95** | **0.82** | **1.01** | **1.03** | **1.00** | **0.94** | **1.08** | **1.23** |
| **1-palmitoylplasmenylethanolamine*** | **1.04** | **0.78** | **1.28** | **0.75** | **0.91** | **1.04** | **0.72** | **1.17** | **0.82** |
| **1-palmitoylglycerol (1-monopalmitin)** | **0.76** | **0.72** | **0.80** | **0.86** | **0.76** | **0.72** | **1.13** | **1.06** | **0.90** |
| **2-palmitoylglycerol (2-monopalmitin)** | **0.79** | **0.82** | **0.99** | **1.06** | **0.83** | **0.75** | **1.33** | **1.02** | **0.76** |
| **1-stearoylglycerol (1-monostearin)** | **0.78** | **0.77** | **0.89** | **0.98** | **0.84** | **0.73** | **1.25** | **1.08** | **0.82** |
| **1-oleoylglycerol (1-monoolein)** | **0.79** | **0.65** | **0.86** | **0.91** | **0.65** | **0.77** | **1.15** | **1.00** | **0.89** |
| **1,3-dipalmitoylglycerol** | **0.79** | **0.77** | **1.01** | **0.87** | **0.77** | **0.68** | **1.10** | **1.01** | **0.67** |
| **acetylcholine** | **0.87** | **0.95** | **0.82** | **1.02** | **1.13** | **1.03** | **1.18** | **1.19** | **1.25** |
| **sphingosine** | **0.72** | **0.67** | **0.74** | **0.79** | **0.83** | **0.80** | **1.10** | **1.24** | **1.08** |
| **palmitoyl sphingomyelin** | **0.85** | **0.98** | **0.96** | **0.99** | **0.85** | **1.20** | **1.16** | **0.87** | **1.25** |
| **stearoyl sphingomyelin** | **0.83** | **1.07** | **0.98** | **0.99** | **0.84** | **0.73** | **1.19** | **0.78** | **0.75** |
| **lathosterol** | **0.67** | **0.56** | **0.64** | **0.58** | **0.57** | **0.85** | **0.87** | **1.01** | **1.33** |
| **cholesterol** | **0.86** | **0.84** | **0.95** | **0.88** | **0.78** | **0.78** | **1.03** | **0.94** | **0.83** |
| **dihydrocholesterol** | **0.86** | **0.64** | **0.93** | **0.80** | **0.77** | **0.79** | **0.92** | **1.20** | **0.84** |
| **24(S)-hydroxycholesterol** | **1.02** | **1.05** | **0.90** | **0.93** | **0.97** | **0.90** | **0.91** | **0.93** | **1.00** |
| **corticosterone** | **0.99** | **1.35** | **12.16** | **0.70** | **1.35** | **1.85** | **0.70** | **1.00** | **0.15** |
| **desmosterol** | **0.69** | **0.75** | **0.80** | **0.63** | **0.55** | **0.91** | **0.92** | **0.74** | **1.14** |
| **xanthine** | **1.03** | **0.93** | **1.10** | **1.12** | **0.74** | **1.13** | **1.09** | **0.80** | **1.03** |
| **hypoxanthine** | **1.17** | **1.17** | **1.05** | **1.16** | **1.37** | **0.96** | **0.99** | **1.17** | **0.91** |
| **inosine** | **1.04** | **0.92** | **0.87** | **1.04** | **0.97** | **0.81** | **1.00** | **1.04** | **0.93** |
| **inosine 5'-monophosphate (IMP)** | **1.15** | **1.00** | **0.97** | **1.12** | **1.16** | **1.00** | **0.97** | **1.16** | **1.03** |
| **adenine** | **1.07** | **1.02** | **1.16** | **1.11** | **1.07** | **1.03** | **1.04** | **1.05** | **0.88** |
| **adenosine** | **1.09** | **0.96** | **0.95** | **1.08** | **0.94** | **0.89** | **0.99** | **0.98** | **0.93** |
| **N1-methyladenosine** | **1.06** | **1.04** | **1.04** | **1.02** | **0.98** | **1.03** | **0.96** | **0.94** | **1.00** |
| **adenosine 5'-monophosphate (AMP)** | **1.01** | **1.04** | **1.06** | **1.13** | **1.02** | **0.99** | **1.12** | **0.99** | **0.93** |
| **adenosine 5'-diphosphate (ADP)** | **1.30** | **1.46** | **1.44** | **1.37** | **1.32** | **1.61** | **1.05** | **0.91** | **1.12** |
| **adenosine 3',5'-cyclic monophosphate (cAMP)** | **0.85** | **0.91** | **0.89** | **0.91** | **1.07** | **0.86** | **1.08** | **1.18** | **0.96** |
| **adenylosuccinate** | **1.21** | **1.47** | **1.31** | **1.32** | **1.26** | **1.05** | **1.09** | **0.86** | **0.80** |
| **guanosine** | **0.94** | **0.87** | **0.84** | **1.01** | **0.85** | **0.70** | **1.08** | **0.98** | **0.83** |
| **guanosine 5'- monophosphate (5'-GMP)** | **1.02** | **1.01** | **1.01** | **1.05** | **1.02** | **1.01** | **1.03** | **1.01** | **1.00** |
| **cytidine** | **1.03** | **0.97** | **1.02** | **1.16** | **1.02** | **1.09** | **1.12** | **1.05** | **1.07** |
| **cytidine 5'-monophosphate (5'-CMP)** | **1.09** | **1.14** | **1.13** | **1.10** | **1.10** | **1.07** | **1.01** | **0.97** | **0.95** |
| **uracil** | **1.10** | **0.90** | **1.15** | **1.06** | **0.98** | **1.45** | **0.97** | **1.09** | **1.26** |
| **5,6-dihydrouracil** | **1.04** | **1.03** | **1.37** | **0.89** | **0.98** | **1.39** | **0.85** | **0.95** | **1.02** |
| **uridine** | **1.01** | **0.98** | **1.02** | **1.05** | **0.97** | **0.93** | **1.04** | **0.98** | **0.91** |
| **pseudouridine** | **0.99** | **0.97** | **0.99** | **1.05** | **0.99** | **1.13** | **1.06** | **1.02** | **1.14** |
| **uridine monophosphate (5' or 3')** | **1.20** | **1.06** | **1.01** | **1.13** | **1.17** | **1.12** | **0.94** | **1.10** | **1.11** |
| **methylphosphate** | **1.10** | **0.94** | **0.92** | **1.08** | **1.11** | **1.15** | **0.99** | **1.17** | **1.25** |
| **ascorbate (Vitamin C)** | **1.07** | **1.09** | **0.92** | **1.11** | **1.06** | **1.05** | **1.04** | **0.97** | **1.13** |
| **threonate** | **0.72** | **0.67** | **0.49** | **0.71** | **0.95** | **1.42** | **0.99** | **1.42** | **2.91** |
| **nicotinamide** | **1.37** | **1.02** | **1.04** | **0.98** | **0.82** | **0.94** | **0.71** | **0.81** | **0.90** |
| **nicotinamide adenine dinucleotide (NAD+)** | **1.01** | **1.03** | **0.96** | **1.03** | **1.03** | **1.03** | **1.02** | **1.01** | **1.08** |
| **nicotinamide adenine dinucleotide reduced (NADH)** | **1.12** | **1.23** | **1.15** | **1.35** | **0.86** | **0.83** | **1.21** | **0.69** | **0.73** |
| **adenosine 5'diphosphoribose** | **1.35** | **1.17** | **1.08** | **1.11** | **0.92** | **0.93** | **0.82** | **0.79** | **0.86** |
| **pantothenate** | **0.99** | **0.94** | **1.29** | **1.02** | **0.95** | **1.30** | **1.03** | **1.01** | **1.01** |
| **coenzyme A** | **2.42** | **3.27** | **3.09** | **3.02** | **1.72** | **1.51** | **1.25** | **0.53** | **0.49** |
| **3'-dephosphocoenzyme A** | **1.91** | **2.09** | **2.26** | **2.09** | **1.46** | **1.09** | **1.09** | **0.70** | **0.48** |
| **acetyl CoA** | **1.47** | **2.41** | **2.45** | **2.19** | **1.58** | **1.63** | **1.49** | **0.65** | **0.66** |
| **flavin adenine dinucleotide (FAD)** | **1.19** | **1.29** | **1.21** | **1.35** | **1.19** | **1.20** | **1.13** | **0.92** | **0.99** |
| **riboflavin (Vitamin B2)** | **0.93** | **0.86** | **0.97** | **1.04** | **0.88** | **1.04** | **1.11** | **1.02** | **1.07** |
| **alpha-tocopherol** | **0.77** | **0.76** | **0.83** | **0.78** | **0.73** | **0.74** | **1.02** | **0.96** | **0.89** |
| **hippurate** | **1.05** | **1.05** | **1.16** | **1.14** | **1.84** | **1.05** | **1.08** | **1.76** | **0.90** |
| **catechol sulfate** | **1.17** | **1.37** | **0.90** | **1.37** | **2.60** | **1.01** | **1.17** | **1.90** | **1.13** |
| **benzoate** | **0.85** | **0.64** | **0.74** | **0.74** | **0.80** | **0.82** | **0.87** | **1.26** | **1.11** |
| **glycolate (hydroxyacetate)** | **1.13** | **1.13** | **0.96** | **1.13** | **1.11** | **1.26** | **1.00** | **0.98** | **1.32** |
| **glycerol 2-phosphate** | **0.92** | **0.99** | **0.62** | **0.95** | **0.95** | **0.80** | **1.04** | **0.96** | **1.29** |
| **2-pyrrolidinone** | **0.98** | **0.92** | **0.78** | **1.04** | **0.71** | **0.78** | **1.06** | **0.77** | **0.99** |
| **Isobar: 2-propylpentanoate, 2-ethylhexanoate** | **0.72** | **0.70** | **0.85** | **0.83** | **0.76** | **0.72** | **1.15** | **1.09** | **0.84** |
| **pentobarbital** | **1.14** | **0.96** | **1.03** | **1.15** | **0.93** | **0.67** | **1.01** | **0.97** | **0.66** |
| **ergothioneine** | **1.11** | **1.06** | **1.11** | **1.10** | **1.05** | **2.37** | **1.00** | **0.99** | **2.13** |
| **stachydrine** | **1.34** | **1.33** | **0.32** | **1.10** | **1.22** | **2.51** | **0.83** | **0.91** | **7.96** |
| **homostachydrine*** | **0.99** | **0.96** | **0.69** | **1.02** | **0.93** | **1.18** | **1.03** | **0.97** | **1.72** |
| **erythritol** | **0.92** | **0.96** | **0.94** | **1.00** | **0.89** | **0.70** | **1.09** | **0.93** | **0.75** |
