# Supplementary material for: Limited Brain Metabolism Changes Differentiate between the Progression and Clearance of Rabies Virus
Source: PLoS One. 2014 Apr 24;9(4):e87180. doi: 10.1371/journal.pone.0087180 (PMC3998930; doi:10.1371/journal.pone.0087180)
Supplement: Table S2 — PET treatment with metyrapone has no effect on WT RABV replication in the CNS over the first 8 days of infection. (DOCX) [file pone.0087180.s005.docx]

**Table S2. PET treatment with metyrapone has no effect on WT RABV replication in the CNS over the first 8 days of infection.**

| Day post infection | DOG4 + Mock | DOG4 + Metyrapone |
| --- | --- | --- |
| 4 | 0.0 ± 0 | 0.0 ± 0 |
| 6 | 27.6 ± 27.0 | 39.3 ± 23.6 |
| 8 | 936.0 ± 573.0 | 1119.0 ± 363.0 |

Mice were infected with 10^4^ FFU of DOG4 RABV i.n. and either mock-treated with PBS or treated with metyrapone (100mg/kg) i.p. each day. Ten mice per group were euthanized at indicated time point, and the number of DOG4 N mRNA copies in brain was quantified by qRT-PCR as described in Materials and Methods. The results are presented as the mean RABV N mRNA copy numbers (+/-SE) per 1,000 copies of L13 mRNA
