# Supplementary material for: Limited Brain Metabolism Changes Differentiate between the Progression and Clearance of Rabies Virus
Source: PLoS One. 2014 Apr 24;9(4):e87180. doi: 10.1371/journal.pone.0087180 (PMC3998930; doi:10.1371/journal.pone.0087180)
Supplement: Table S3 — The metyrapone-treated mice that survived WT RABV infection develop significant serum RABV-specific neutralizing antibodies (VNA) titers. (DOCX) [file pone.0087180.s006.docx]

**Table S3. The metyrapone-treated mice that survived WT RABV infection develop significant serum RABV-specific neutralizing antibodies (VNA) titers.**

| Day post infection | DOG4 + Mock | DOG4 + Metyrapone |
| --- | --- | --- |
| 8 | 0.07 (n=10) | 0.00 (n=10) |
| 10 | ND | 0.00 (n=6) |
| 12 | ND | 1.43 (n=6) |

Mice were infected with 10^4^ FFU of DOG4 RABV i.n. and either mock-treated with PBS or treated with metyrapone (100mg/kg) i.p. each day. Animals were bled at each time point 1 hr after treatment. Serum VNA titers were determined by the rapid fluorescent focus inhibition test and are presented as the geometric mean international units (IU). ND – not done.
